# An Inter‐Supplementary Biohybrid System Based on Natural Killer Cells for the Combinational Immunotherapy and Virotherapy of Cancer

**DOI:** 10.1002/advs.202103470

**Published:** 2021-11-07

**Authors:** Li Ding, Qingqing Gao, Zhuobin Xu, Liangliang Cai, Sujuan Chen, Xinyue Zhang, Peng Cao, Gang Chen

**Affiliations:** ^1^ College of Bioscience and Biotechnology Yangzhou University Yangzhou Jiangsu 225009 P. R. China; ^2^ College of Veterinary Medicine Yangzhou University Yangzhou Jiangsu 225009 P. R. China; ^3^ Institute of Comparative Medicine Yangzhou University Yangzhou Jiangsu 225009 P. R. China; ^4^ Jiangsu Co‐innovation Center for Prevention and Control of Important Animal Infectious Diseases and Zoonoses Yangzhou University Yangzhou Jiangsu 225009 P. R. China; ^5^ Institute of Translational Medicine Medical College Yangzhou University Yangzhou Jiangsu 225009 P. R. China; ^6^ Affiliated Hospital of Integrated Traditional Chinese and Western Medicine School of Pharmacy Nanjing University of Chinese Medicine Nanjing Jiangsu 210023 P. R. China

**Keywords:** anticancer immunity, cell carriers, natural killer cells, oncolytic adenovirus, tumor‐targeted delivery

## Abstract

Oncolytic adenoviruses (Ads) have gained great attention in cancer therapy because they cause direct cytolytic infection and indirectly induce antitumor immunity. However, their efficacy is compromised by host antiviral immune response, poor tumor delivery, and the immunosuppressive tumor microenvironment (TME). Here, a natural killer (NK) cell‐mediated Ad delivery system (Ad@NK) is generated by harnessing the merits of the two components for combinational immunotherapy and virotherapy of cancer. In this biohybrid system, NK cells with a tumor‐homing tropism act as bioreactors and shelters for the loading, protection, replication, amplification, and release of Ads, thereby leading to a highly efficient systemic tumor‐targeted delivery. As feedback, Ad infection offers NK cells an enhanced antitumor immunity by activating type I interferon signaling in a STAT4‐granzyme B‐dependent manner. Moreover, it is found that the Ad@NK system can relieve immunosuppression in the TME by promoting the maturation of dendritic cells and the polarization of macrophages to M1 phenotype. Both in vitro and in vivo data indicate the excellent antitumor and antimetastatic functions of Ad@NKs by destroying tumor cells, inducing immunogenic cell death, and immunomodulating TME. This work provides a clinical basis for improved oncolytic virotherapy in combination with NK cell therapy based on the inter‐supplementary biohybrid system.

## Graphical abstract

A natural killer cell‐based oncolytic adenovirus delivery system is developed by harnessing the merits of the two components for combinational immunotherapy and virotherapy of triple‐negative breast cancer. The powerful therapeutic efficacy of this system against the development and metastasis of tumors demonstrates the great translational potential of this combinational strategy.

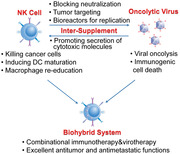

## 1 Introduction

Oncolytic virotherapy is currently considered an alternative for cancer patients who do not respond or fail to achieve durable responses to immune checkpoint inhibitors.^[^ ^1^ ^]^ Oncolytic adenoviruses (Ads), which display tumor‐selective replicating and cytopathic effects, have been extensively explored for the treatment of malignant neoplasms.^[^ ^2^, ^3^ ^]^ Cumulative viral replication results in tumor cell lysis and antigen release in the tumor microenvironment (TME), followed by triggering both the innate and adaptive immune responses.^[^ ^4^ ^]^ To date, several clinical trials have shown the efficacious use of oncolytic viruses in localized cancer therapy.^[^ ^5^ ^]^ However, the tumor‐targeting efficiency of Ads after intravenous administration is compromised by the immunologic barriers in the blood system including nonspecific sequestration, antiviral immunity, and the innate immune response.^[^ ^6^ ^]^ Even worse, the immunosuppressive mechanisms and lack of sufficient immunopositive cells in the nonimmunogenic “cold” TME limit the efficacy of Ads.^[^ ^7^ ^]^

To overcome these obstacles, many types of engineered biomaterials, such as lipids,^[^ ^8^ ^]^ polymers,^[^ ^9^ ^]^ cell membranes,^[^ ^10^ ^]^ and extracellular vesicles,^[^ ^11^ ^]^ have been developed as systemic delivery tools to protect Ads from immune clearance in the blood.^[^ ^12^ ^]^ In recent studies, tumor‐homing cells such as mesenchymal stem cells (MSCs) have received considerable attention because of their inherent advantages as bioreactors for the replication and amplification of Ads as an enhancement to the amount of Ads delivered, rather than just as vehicles.^[^ ^13^, ^14^, ^15^ ^]^ Although there are many preclinical studies on the MSC‐based delivery system as a potential cancer virotherapy strategy, MSC therapy is still stagnated in its infancy because of its potential contribution to tumor progression. As reported, MSCs can transform into malignant cells or more mature mesenchymal cells such as cancer‐associated fibroblasts due to the induction of tumor‐promoting factors secreted by tumor cells.^[^ ^16^, ^17^ ^]^ Besides, MSCs can also construct an immunosuppressive environment in the TME by recruiting myeloid‐derived suppressor cells to promote tumor growth.^[^ ^17^ ^]^ Furthermore, the accelerated epithelial‐mesenchymal transition (EMT) induced by MSCs indicates an increased risk of tumor metastasis in clinical applications.^[^ ^17^, ^18^, ^19^ ^]^ Apart from the above challenges, the lack of intrinsic anticancer properties is another limitation for the application of MSCs in tumor treatment.^[^ ^13^ ^]^ In contrast, natural killer (NK) cells are highly cytotoxic immune effectors, which can generate and release cytotoxic molecules to directly destroy tumor cells.^[^ ^20^ ^]^ More importantly, NK cells have the ability to educate M2 macrophages to pro‐inflammatory M1‐type macrophages in the TME, facilitate the maturation of antigen‐presenting cells (APCs), and in turn, stimulate T cells to attack tumor cells. In addition, as one of the members of the innate immune system, NK cells have a well‐established homing ability to accumulate in tumor sites. Collectively, NK cells present another solution for generating an active tumor‐targeting delivery system as a replacement for MSCs.^[^ ^21^ ^]^

**Scheme 1:**
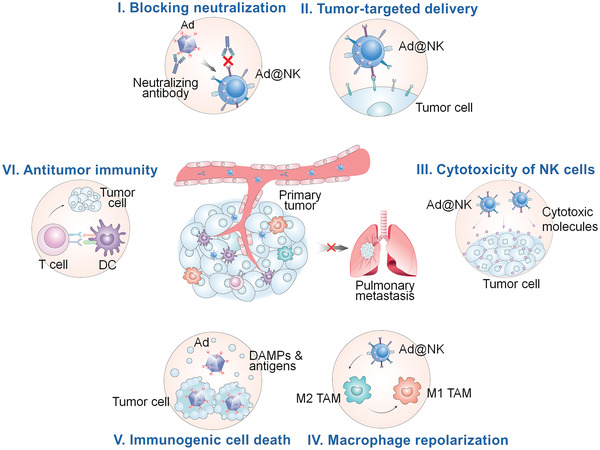
Schematic of proposed mechanism performed by Ad@NKs for the suppression function against primary tumor and accompanying metastatic dissemination. I) NK cells protect inside Ads from immune clearance by blocking the antibody neutralization in systemic circulation. II) NK cells act as bioreactors and drug carriers for the loading, replication, amplification and tumor‐targeted delivery of Ads. III) NK cells are powerful immune effectors by releasing cytotoxic molecules, which displays a synergistic effect with Ads to kill tumor cells. IV) Ad@NKs promote the repolarization of M2 macrophages to the M1 ones, resulting in an enhanced antitumor immunity. V) Ads induce the lysis of the host tumor cells, which in turn release a mass of damage‐associated molecular patterns (DAMPs) and tumor antigens for an immune activation against cancer. VI) Ad@NKs accelerate the maturation of DCs thereby activating cytotoxic CD8^+^ T lymphocytes (CTLs) against tumor cells.

In the present study, we harnessed the merits of oncolytic Ads and NK cells to develop a biohybrid system named Ad@NK. In this system, the properties for the loading, protection, amplification, and release of Ads by NK cells were optimized to boost their oncolytic activity and antitumor immunity, thereby leading to a synergistic interplay between these two components. After systemic administration, Ad@NKs conducted a potent tumor‐targeted virotherapy in combination with immunotherapy in triple‐negative breast cancer (TNBC) models (**Scheme** **1**). The powerful therapeutic efficacy of Ad@NKs against the development and metastasis of tumors was corroborated by the in vitro and in vivo data, demonstrating the great translational potential of this combinational strategy.

## 2 Results

### 2.1 Ads Show a Tumor‐Selective Replication and Cytotoxicity

**Figure 1:**
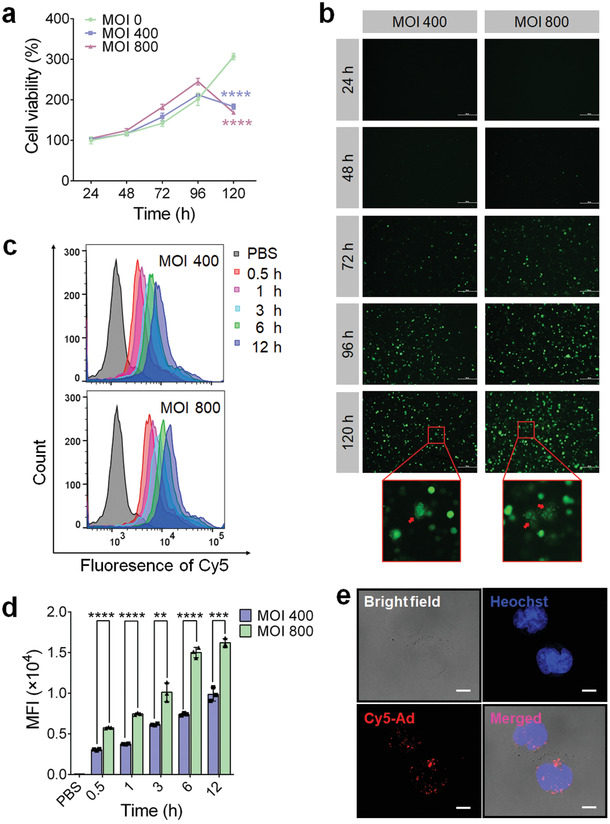
NK cells are applicable carriers for the loading of Ads. a) CCK8 assay was performed to assess the Ad‐mediated cytotoxicity to NK cells. b) After different incubation times at MOI 400 or 800, the EGFP fluorescence indicating the transcription and expression of virogenes in NK cells was imaged under a fluorescence microscope. Scale bars: 50 µm. c,d) Cellular uptake by NK cells that determined by flow cytometry after different time points post the co‐incubation with Cy5‐labelled Ads at MOI 400 or 800. e) Confocal microscope images of NK cells after an incubation with Cy5‐labelled Ads (red) for 12 h. Cell nuclei were stained with DAPI (blue). Scale bars: 10 µm. Data are represented as mean ± SD, *n* = 3. **P < 0.01; ****P < 0.0001 denote significant difference.

To generate a tumor‐selective Ad, a 24‐bp fragment was removed from the E1A gene of human Ad type 5 (Figure S1, Supporting Information).^[^ ^22^ ^]^ In addition, an EGFP‐coding DNA, as an indicator for the transcription and replication of virogenes, was inserted into the genome of Ads (Figure S1, Supporting Information). After packaging, purification and titration, the Ads were added to mouse NK cells and 4T1 tumor cells to investigate their cytotoxicity using the CCK8 assay. As shown in **Figure** **1**a, Ads showed no significant cytotoxicity to NK cells until 96 h post treatment at a multiplicity of infection (MOI) of 400 or 800, which indicates the ratio of infectious viral particles (PFU)/cell in the co‐incubation system. In contrast, at a much lower dose (MOI 0.3125) of Ads, the proliferation of 4T1 cells showed a remarkable inhibition at 72 h post treatment (*P* < 0.001) (Figure S2, Supporting Information). Cytotoxicity increased with increasing doses of Ads and incubation time (Figure S2, Supporting Information). Moreover, EGFP fluorescence was observed in 4T1 cells starting at 24 h post treatment (MOI 5 or 10) (Figure S3a, Supporting Information), whereas visible fluorescence signals in the NK cells could not be captured within 48 h at much higher doses of Ads (MOI 400 or 800) (Figure 1b). Consistent with this, the infection cycle of Ads was 72 h in NK cells, four times greater than that (18 h) in 4T1 cells (Figure S4, Supporting Information), suggesting a faster replication rate of Ads in tumor cells than in normal NK cells.

In addition, we compared the fluorescence intensity and cytotoxicity in different normal and tumor cells from different species. Remarkable EGFP fluorescence (Figure S3, Supporting Information) and cytotoxicity (Figure S5, Supporting Information) were observed in 4T1 (mouse) and MDA‐MB‐231 (human) TNBC cells treated with 10 MOI of Ads, but in neither L929 mouse fibroblasts nor Nrk‐52e rat renal proximal tubular cells, indicating a much higher replication rate and cytotoxicity of Ads in tumor cells than in normal cells. Due to the high similarity (≈90% in protein sequence) of human (NM_001338.5) and mouse (NM_001025192.3) coxsackievirus and adenovirus receptor (CXADR) (Figure S6, Supporting Information), Ads led to a similar infectivity to 4T1 and MDA‐MB‐231 cells via CXADR‐mediated cellular uptake ^[^ ^23^ ^]^ (Figure S7, Supporting Information), and a similar replication rate and cytotoxicity in the two tumor cells (Figures S3 and S5, Supporting Information), regardless of species.

Taken together, the above data showed that Ads had a much higher replication efficiency and cytotoxicity in tumor cells than in NK or other normal cells, revealing a tumor‐selective suppression function of Ads used in this study.

### 2.2 NK Cells are Feasible Carriers and Bioreactors for the Loading, Replication, Amplification, and Protection of Ads

**Figure 2:**
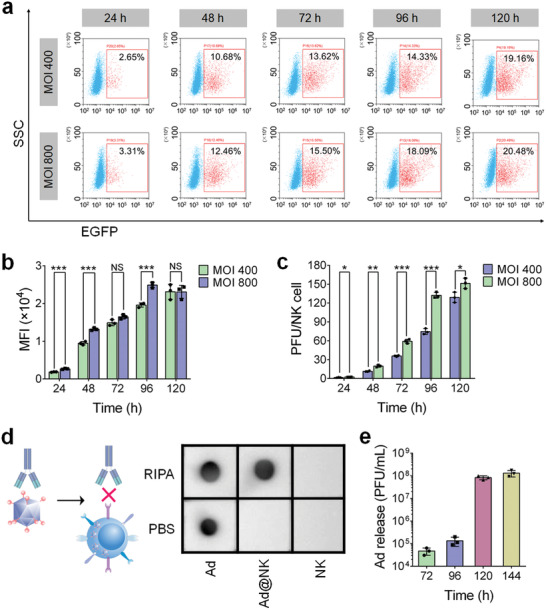
NK cells mediate an efficient replication, protection and release of Ads. a) The positive rates and b) mean fluorescence intensity (MFI) of EGFP in NK cells were quantitated by FACS analysis, after treated with Ads at MOI 400 or 800 for different times. c) The mean number of PFU in per NK cell, at particular intervals post Ad treatment, was tittered in a reference cell line 293A. d) Dot blot assay using an anti‐Adenovirus hexon antibody was carried out to evaluate the protective effects of NK cells on inside Ads by blocking antibody binding. e) After an incubation with Ads at MOI 800 for 48 h, 7 × 10^5^ NK cells were moved to 1 mL fresh medium, in which the release Ads were collected and tittered at different time points post Ad infection. Data are represented as mean ± SD, *n* = 3. *P < 0.05; **P < 0.01; ***P < 0.001 denote significant difference. NS, not significant versus the relevant group.

To confirm the feasibility of constructing a biohybrid system based on NK cells and Ads, we first explored whether NK cells are suitable carriers for Ads by evaluating the Ad loading capacity of NK cells via cellular uptake. As shown in Figure 1c,d, and Figure S8 (Supporting Information), the uptake of Cy5‐labeled Ads (Cy5‐Ads) by NK cells increased within 12 h in a time‐ and dose‐dependent manner. Consistent with the flow cytometry results, the fluorescence images taken via confocal microscopy further corroborated efficient cell uptake, which is indicated by the strong intracellular distribution of the red fluorescence of Cy5 in NK cells (Figure 1e). We then evaluated the cellular replication levels of Ads by measuring the EGFP fluorescence intensity at different time points post treatment, and found that the percentage of EGFP‐positive cells and the cellular mean fluorescence intensity (MFI) increased continuously until 120 h in a dose‐dependent manner (**Figures** 1b and **2**a,b). Afterward, the Ads accumulated in the cells were extracted through repeated freeze‐thawing, and were tittered in a reference cell line 293A. Similar to the variation trend of EGFP fluorescence intensity, the intracellular PFU also increased significantly with an increase in incubation time in a dose‐dependent manner (Figure 2c), indicating that NK cells were not merely carriers, but also acted as bioreactors for the replication and amplification of Ads, thereby leading to an enhanced loading.

Because the replication and amplification of Ads in NK cells showed a sharp increase at 48 h (Figures 1b and 2a,b) and reached saturation point prior to cell death at a time point between 96 and 120 h after Ad treatment (Figures 1a,b and 2a,b), we used an optimized incubation time of 48 h at MOI 800 for the preparation of Ad@NKs (≈20 PFU per cell) (Figure 2c) to balance the viral dose and the immune activity of NK cells.

Furthermore, to investigate the protective function of NK cells for the inside Ads against antibody neutralization, we performed a dot blot assay, and found that the Ads carried by NK cells could not be detected by a specific antibody because of the obstructive function of the cell membranes (Figure 2d), suggesting that NK cells had the ability to provide Ads with a shelter for blocking the antibody neutralization effect mediated by humoral immunity.^[^ ^24^ ^]^ In contrast, once the cells were lysed using RIPA buffer, the binding between antibodies and Ads were restored due to the removal of the membrane‐mediated hindrance (Figure 2d).

Therefore, NK cells were suitable hosts for the loading, replication, and amplification of Ads, and provided a refuge for their cargos by cutting off antigen‐antibody interactions.

### 2.3 Ad‐Mediated Cell Lysis Leads to an Efficient Ad Release

In the process of monitoring EGFP fluorescence in NK cells after Ad treatment, a dispersive green fluorescence was observed at 120 h (Figure 1b), indicating the initiation of cell lysis induced by Ad accumulation, which was consistent with the sudden drop in the cell viability of NK cells at the same time point (*P* < 0.0001) (Figure 1a). Based on these results, we speculated that a high‐efficiency Ad release started around 120 h post treatment, later than the end of the first life cycle (72 h) of Ads (Figure S4, Supporting Information). To validate this speculation, 7 × 10^5^ NK cells that had already been co‐incubated with Ads (MOI 800, 48 h), containing a total of ≈1.4 × 10^7^ PFU of Ads, were seeded into 1 mL fresh medium, in which the titer of released Ads was determined at the given time points after removing the cells via centrifugation. As shown in Figure 2e, ≈4.7 × 10^4^ and ≈1.1 × 10^5^ PFU of Ads were detected in the medium at 72 and 96 h post Ad treatment, respectively, indicating that only a small portion of Ads was released from NK cells in the first 48 h. At 120 h, ≈8.4 × 10^7^ PFU of Ads were released into the medium, then it increased to ≈1.2 × 10^8^ at 144 h (Figure 2e). The sharp increase corroborated our speculation that Ad release sped up between 96 and 120 h post treatment due to Ad‐induced cell lysis. On the other hand, the amount of released Ads at 120 and 144 h was much higher than the initial amount of Ads carried by NK cells at 48 h, which was mainly attributed to the proliferation of NK cells and the continuous replication of intracellular Ads prior to cell death, further confirming the compatibility between Ads and NK cells.

In summary, Ad‐induced cell lysis and death at a particular time point was conducive to the release of Ads from NK cells, which was a key requirement for the controllable delivery of therapeutic loads.

### 2.4 Ad Loading Augments NK Cell Functions by Upregulating Type I Interferon Signaling Pathways, Involving with STAT4 and Granzyme B

**Figure 3:**
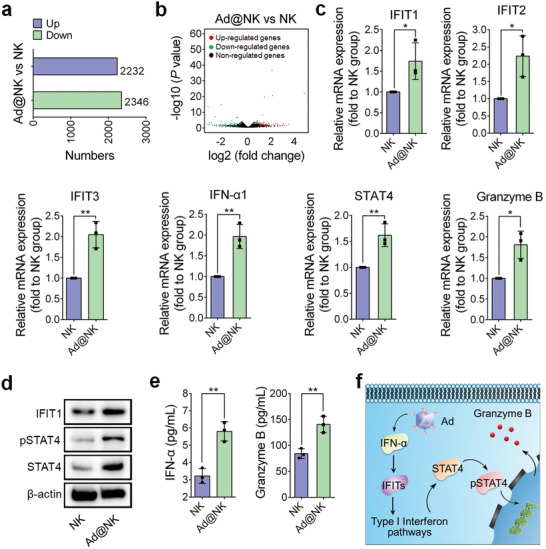
Ad treatment upregulates immunity‐related pathways in NK cells. a,b) Statistics for the number and proportion of the genes upregulated or downregulated in the transcriptome of Ad@NKs versus untreated NK cells. c) qRT‐PCR, d) western blot, and e) ELISA analyses for the expression levels of the differentially expressed genes (DEGs) (Figure S9b, Supporting Information) and the related immunomodulatory genes in Ad@NKs versus in NK cells. f) Schematic of activated signaling pathways in NK cells in response to Ad stimulation. Data are represented as mean ± SD, *n* = 3. *P < 0.05; **P < 0.01 denote significant difference.

As shown in recent studies,^[^ ^25^ ^]^ NK cells show immune responses to viral infections, which suggests us that Ad treatment may be an agonist for the immune activity of NK cells. To verify this hypothesis, we conducted a transcriptome sequencing analysis in Ad‐treated NK cells. As shown in **Figure** **3**a and Figure S9a (Supporting Information), the gene transcriptional profile in Ad@NKs showed a noticeable change compared to that in untreated NK cells, which was further visually expressed by a volcano plot (Figure 3b). Moreover, Gene Ontology (GO) enrichment analysis indicated that most of the top 10 pathways, enriched with differentially expressed genes (DEGs) such as IFIT1, IFIT2, and IFIT3, are highly related to immune regulation (Figure S9b,c: Supporting Information). As reported, IFITs, especially IFIT1, are commonly upregulated in response to increased expression of type I interferon (IFN‐*α* or IFN‐*β*) after virus infection, followed by a strong activation of downstream type I interferon signaling.^[^ ^26^ ^]^ Accordingly, we performed qRT‐PCR to evaluate the mRNA levels of IFN‐*α*1, IFITs, and downstream genes in type I interferon signaling pathways. As shown in Figure 3c, the mRNA levels of IFN‐*α*1 and IFITs increased significantly after Ad infection (*P* < 0.05), thereby upregulating the transcriptional levels of STAT4 (*P* < 0.01), a key effector molecule in type I interferon signaling pathways,^[^ ^27^ ^]^ and its target gene granzyme B (*P* < 0.05) that regulated by phosphorylated STAT4 (pSTAT4).^[^ ^28^ ^]^ Furthermore, the protein levels of IFN‐*α* (*P* < 0.01), IFIT1, STAT4, and pSTAT4 (Tyr693) were all upregulated in response to Ad infection (Figure 3d,e), thereby leading to an increased secretory expression of granzyme B (*P* < 0.01) (Figure 3e), which provided solid evidence to support our hypothesis that Ad loading facilitated NK cell activity in a STAT4‐granzyme B‐dependent manner (Figure 3f).

Overall, NK cells showed a significant immune response to Ad infection by activating type I interferon signaling pathways, in which the transcription, translation, and phosphorylation levels of STAT4 were all upregulated, thereby augmenting the transcription and secretory expression of granzyme B, a proven cytotoxic molecule inducing cancer cell apoptosis.^[^ ^29^ ^]^

### 2.5 Ad@NKs Show Higher In Vitro Anticancer Activity than a Single Treatment with Ads or NK Cells

**Figure 4:**
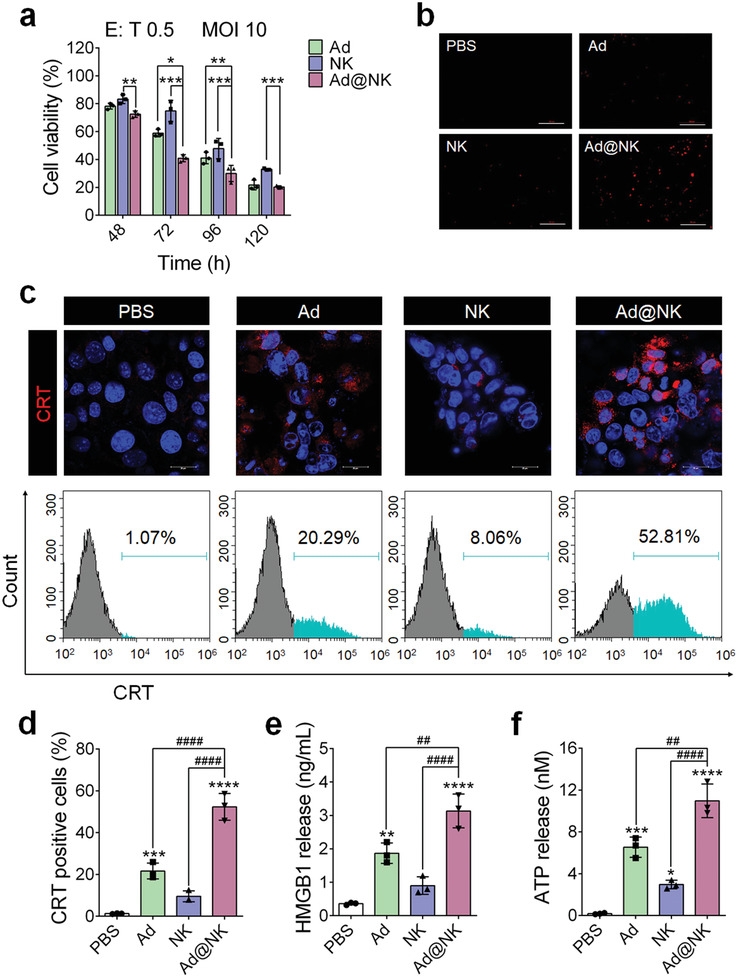
Ad@NKs possess a higher in vitro tumor cell killing effect than NK cells or Ads alone. a) CCK8 assay was performed to evaluate the cell viability of 4T1 after treatment of Ads (MOI 10), NK cells (E: *T* = 0.5) and Ad@NKs (MOI 10, E: *T* = 0.5), respectively. b) TUNEL assay was performed to evaluate the apoptosis of 4T1 cells induced by the treatments of Ads, NK cells or Ad@NKs. Scale bars: 100 µm. c, d) After the treatments of Ads, NK cells or Ad@NKs, the immunofluorescence marking surface‐exposed CRT (red) on 4T1 cells was recorded by laser confocal microscope and flow cytometry detection. Cell nuclei were stained with DAPI (blue). Scale bars: 20 µm. e) ELISA and f) ATP assays were performed to determine the amounts of HMGB1 protein and ATP molecules, respectively, which were released from the 4T1 cells treated with Ads, NK cells or Ad@NKs. Data are represented as mean ± SD, *n* = 3. *P < 0.05; **P < 0.01; ***P < 0.001; ****P < 0.0001 (vs indicated or PBS); ^##^ *P* < 0.01; ^####^ *P* < 0.0001 (vs indicated) denote significant difference.

To evaluate the anticancer efficacy of Ad@NKs in vitro, we first performed a CCK8 assay and found that Ad@NK treatment induced a higher cytotoxicity to 4T1 tumor cells in comparison with treatments of NK cells or Ads alone in a time‐ and dose‐dependent manner (**Figure** **4**a and Figure S2, Supporting Information). The combination index (CI) of Ads and NK cells was calculated to 0.32 < 1, indicating an obvious synergistic effect.^[^ ^30^ ^]^ After 72 h treatment (MOI 10, effector‐to‐target ratio (E: T) = 0.5), the viability rate of 4T1 cells treated with Ad@NKs was 40.9 ± 2.5% in comparison with the control at 0 h, a significant reduction compared to the cells treated with Ads (58.9 ± 2.7%) (*P* < 0.05) or NK cells (74.8 ± 7.6%) (*P* < 0.001) (Figure 4a). Moreover, the results of the TUNEL assay also indicate a higher cell apoptosis rate induced by Ad@NKs than that by NK cells or Ads (Figure 4b).

Considering that surface‐exposed calreticulin (CRT), secreted ATP, and released high mobility group protein B1 (HMGB1) are key indicators for immunological cell death (ICD),^[^ ^31^, ^32^ ^]^ partly by which oncolytic viruses trigger the death of host cells,^[^ ^33^ ^]^ we performed cell immunofluorescence, flow cytometry, ELISA, and ATP assays. We found that the levels of all these three indicators in the 4T1 cells subjected to Ad@NK treatment showed significant increases compared to those in the groups treated with Ads, NK cells, or PBS control (Figure 4c–f). As revealed by recent studies,^[^ ^32^, ^34^ ^]^ the lysis of cancer cells succumbing to ICD leads to a large release of damage‐associated molecular patterns (DAMPs), which can activate the immune system to strengthen the recognition of cancer cells, thereby making an extra contribution to Ad@NK‐induced cytotoxicity.

Overall, our data demonstrated the superiority of Ad@NKs over single treatment with Ads or NK cells to induce an in vitro killing effect on cancer cells by triggering apoptosis and ICD.

### 2.6 NK Cells Show a High‐Efficiency Tumor Homing Tropism for the Targeted Delivery of Ads

**Figure 5:**
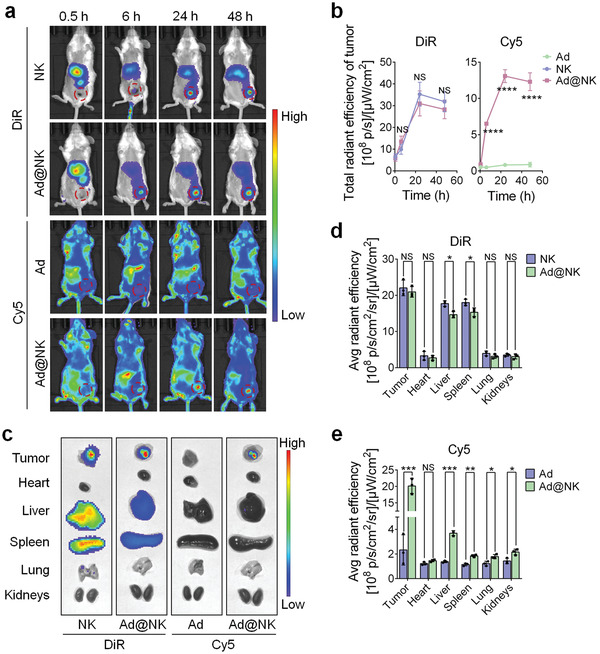
NK cells exhibit an excellent tumor homing ability for the tumor‐targeted delivery of Ads. a) Whole‐body biodistribution of Ads, NK cells, and Ad@NKs in 4T1‐bearing mice at different time points post intravenous administration. Ads were labelled with Cy5 (Cy5‐Ads) and NK cells were labelled with DiR (DiR‐NK). b) The total radiant efficiencies of different tumors were recorded and analyzed using IVIS Living imaging software. c) Fluorescent images were taken for the tumor tissues and main organs (heart, liver, spleen, lung, and kidneys) which were harvested from the sacrificed mice at 48 h post different treatments, d, e) followed by calculating the average radiant efficiencies of theses tissues. Data are represented as mean ± SD, *n* = 3. *P < 0.05; **P < 0.01; ***P < 0.001; ****P < 0.0001 denote significant difference. NS, not significant versus the relevant group.

To identify the tumor homing capacity of NK cells, DiR dye was used to label the cell membranes for a comprehensive in vivo biodistribution study in tumor‐bearing mice. The PBS‐treated mice (blank control) showed no background fluorescence in the whole body and main organs under the detectable waveband of DiR, which ruled out the background interference (Figure S10, Supporting Information). At 6 h post intravenous injection, noticeable fluorescence signals of DiR were observed in the tumors of the mice treated with NK cells or Ad@NKs, confirming the high affinity between NK cells and tumor tissues (**Figure** **5**a). The tumor accumulation of NK cells peaked at 24 h and showed a slight decline at 48 h post injection (Figure 5a). As expected, Ad loading did not alter the targeting ability of NK cells (Figure 5a,b). To address whether the homing ability of NK cells contributes to tumor‐targeted delivery, Cy5 dye was conjugated to the Ad capsid protein to investigate the biodistribution of Ads carried by NK cells. Under the detectable waveband of Cy5, the PBS‐treated mice showed a relative high fluorescence in the stomach and intestines, but a very low level of background interference in the rest body regions (Figure S10, Supporting Information). From 6 to 48 h post injection, the Cy5 fluorescence showed a similar biodistribution and tumor accumulation to the DiR fluorescence of NK cells, indicating that NK cells transported Ads to the tumor sites at a high efficiency (Figure 5a,b). The number of intratumor Ad copies continuously increased by replication until day 6 post injection, and then began a slow decline (Figure S11, Supporting Information). In contrast, free Cy5‐Ads showed no tumor targeting ability (Figure 5a,b). At 48 h post injection, we harvested the tumor tissues and main organs from the sacrificed mice to measure the average fluorescence intensity of Cy5 and DiR, and found that the cells loading with Ads showed a similar distribution to empty NK cells in tumors and the main organs, whereas free Ads showed a much lower accumulation in tumors and all the main organs, except the heart, than that carried by NK cells (Figure 5c–e).

To assess the drug release kinetics, the number of Ad particles was measured in the serum of mice after treatment with Ad@NKs. The in vivo release of Ads started at 12–24 h post injection, and increased to the peak at 48 h (Figure S12a, Supporting Information). This result indicated that Ad release did not start until NK cells arrive at tumor sites (6 h), where most of Ads were released from 12 to 48 h. 72 h post injection, the number of released Ads began to decline (Figure S12a, Supporting Information). To investigate the pharmacokinetic parameters, the number of Ads was also measured in the whole blood. Compared with free Ads (0.54 ± 0.28 h), the in vivo half‐life (*t*_1/2_) of Ads carried by NK cells was extended to 5.07 ± 0.95 h (Figure S12b, Supporting Information), suggesting that NK‐based drug loading was conducive to prolong the action time of Ads.

In short, these data indicated that the highly efficient targeted delivery of Ads was attributed to the tumor‐homing ability of NK cells. Further, Ad loading had no significant effects on the tumor‐homing tropism of NK carriers.

### 2.7 Ad@NKs Show a Stronger Curative Effect than Ads or NK Cells Alone for Tumor Growth and Metastasis in Mice with TNBC

**Figure 6:**
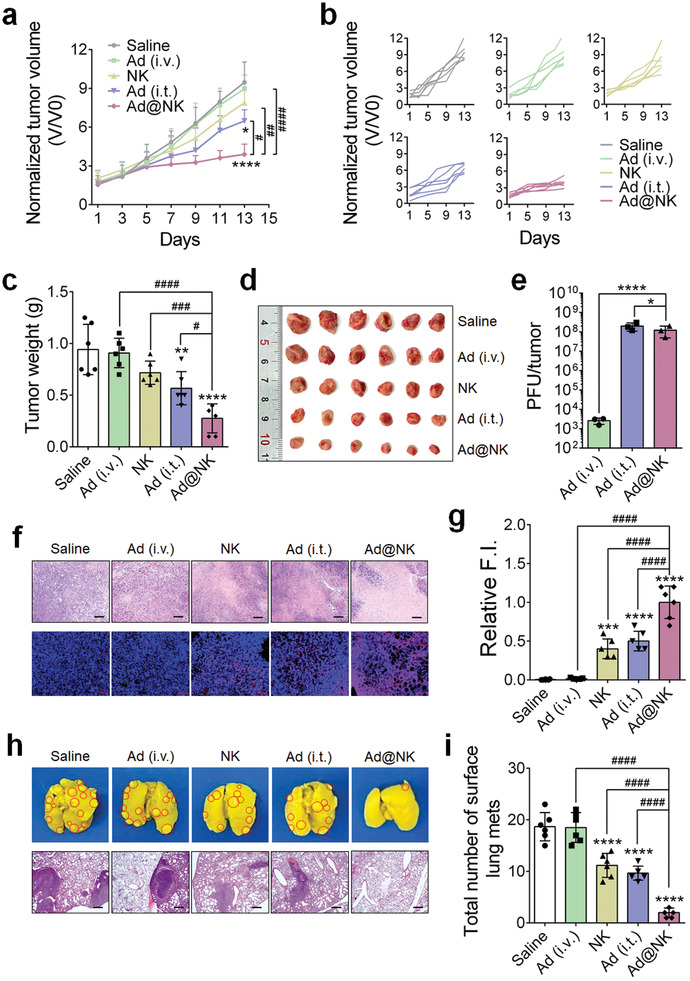
Ad@NK treatment shows a stronger in vivo inhibitory activity for the primary and metastatic tumors than either of Ad or NK cell single treatment in 4T1‐bearing mice. a) Average or b) individual tumor growth curves in different treatment groups (*n* = 6). c) Mean tumor weights and d) images of primary tumors in different treatment groups at the end of a 14‐d course of treatment (*n* = 6). e) Mean numbers of PFU in per tumor in Ad (i.v.), Ad (i.t.) and Ad@NK treatment groups at day 9 post injection (*n* = 3). f) Representative photographs for the tumor sections which were stained by H&E (scale bars: 100 µm) or TUNEL (scale bars: 50 µm). Cell nucleus: blue; TUNEL: red. g) Relative fluorescence intensity (F.I.) of TUNEL in different treatment groups at day 14 (*n* = 6). h) Representative photographs for the whole lung tissues and H&E staining of lung sections. Red circles denote metastases. Scale bars: 200 µm. i) Mean number of surface lung metastases at day 14 (*n* = 6). Data are represented as mean ± SD. *P < 0.05; **P < 0.01; ***P < 0.001; ****P < 0.0001 (vs indicated or Saline); ^#^ *P* < 0.05; ^##^ *P* < 0.01; ^###^ *P* < 0.001; ^####^ *P* < 0.0001 (vs indicated) denote significant difference.

To evaluate the in vivo therapeutic efficacy of Ad@NKs, we constructed mice models with TNBC by injecting 4T1 cells into the fat pads of mice. In these models, treatments with Ad@NKs (intravenous injection, i.v.) and Ads (intratumor injection, i.t.) exhibited inhibition rates of ≈60% (*P* < 0.0001) and ≈30% (*P* < 0.05), respectively, in the growth of primary tumors in comparison with the saline‐treated controls at the end of the treatment course (**Figure** **6**a). However, the tumor growth rates, mean tumor volumes and tumor weights in either Ads (i.v.) or NK cells (i.v.) treatment groups were not significantly different from those in the saline‐treated group (Figure 6a–c). Among the different single dosage regimens, Ad@NKs showed the highest tumor inhibition effect, especially a fold higher antigrowth rate than that induced by Ads (i.t.) (*P* < 0.05) (Figure 6a–c), which was further visually verified through the photographed tumors excised from the sacrificed mice (Figure 6d). Moreover, the tumor growth rate in Ad@NK treatment group was similar to that in Ads (i.t.) + NKs (i.v.) group and significantly slower than that in Ads (i.v.) + NKs (i.v.) group (*P* < 0.01), indicating a superiority of Ad@NK formulation over direct Ad+NK doublet via intravenous administration in treating tumors (Figure S13, Supporting Information). To explore the relationship between Ad replication and tumor suppression, the PFU of Ads in the primary tumor tissues was measured at day 9 after the different treatments. As shown in Figure 6e, a tumor contained an average of 1.2 × 10^8 ^± 1.3 × 10^7^ PFU of Ad after Ad@NK injection, exponentially higher than that in the Ad (i.v.) treatment group (3.3 × 10^3 ^± 5.1 × 10^2^ PFU/tumor) (*P* < 0.0001) due to the targeted delivery mediated by NK cells for an enhanced virotherapy efficacy, but a little lower than that in the Ad (i.t.) group (1.8 × 10^8 ^± 3.9 × 10^7^ PFU/tumor) (*P* < 0.05). The non‐corresponding levels of Ad replication and tumor inhibition in the two groups indicated that the superiority of Ad@NKs over Ads (i.t.) in cancer treatment was due to the additional contribution of NK cells. Moreover, the advantages of Ad@NK treatment were further verified through the analyses of H&E and TUNEL staining, which indicated the higher rates of necrosis and apoptosis of tumor cells in the mice treated with Ad@NKs in comparison with those in any other single‐drug treatment group (*P* < 0.0001) (Figure 6f,g).

In view of the high incidence of metastasis in clinical cases of TNBC,^[^ ^35^ ^]^ we assessed the antimetastatic efficacy of different treatments in 4T1‐bearing mice by counting the number of surface lung metastases, which is the most common site of tumor metastasis according to previous studies.^[^ ^4^ ^]^ As shown in Figure 6h,i, compared to the saline control, Ad@NK treatment dramatically decreased the number of metastatic lesions (*P* < 0.0001), showing higher antimetastatic efficacy than treatment with Ads (i.t.) (*P* < 0.0001), NK cells (*P* < 0.0001), or Ads (i.v.) (*P* < 0.0001).

In summary, we found that Ad@NKs had higher cancer cell killing and apoptosis induction activity than the other treatments, thereby exhibiting the strongest in vivo antitumor and antimetastatic efficacy for TNBC.

### 2.8 Ad@NKs Induced an Enhanced Antitumor Immunity In Vivo

**Figure 7:**
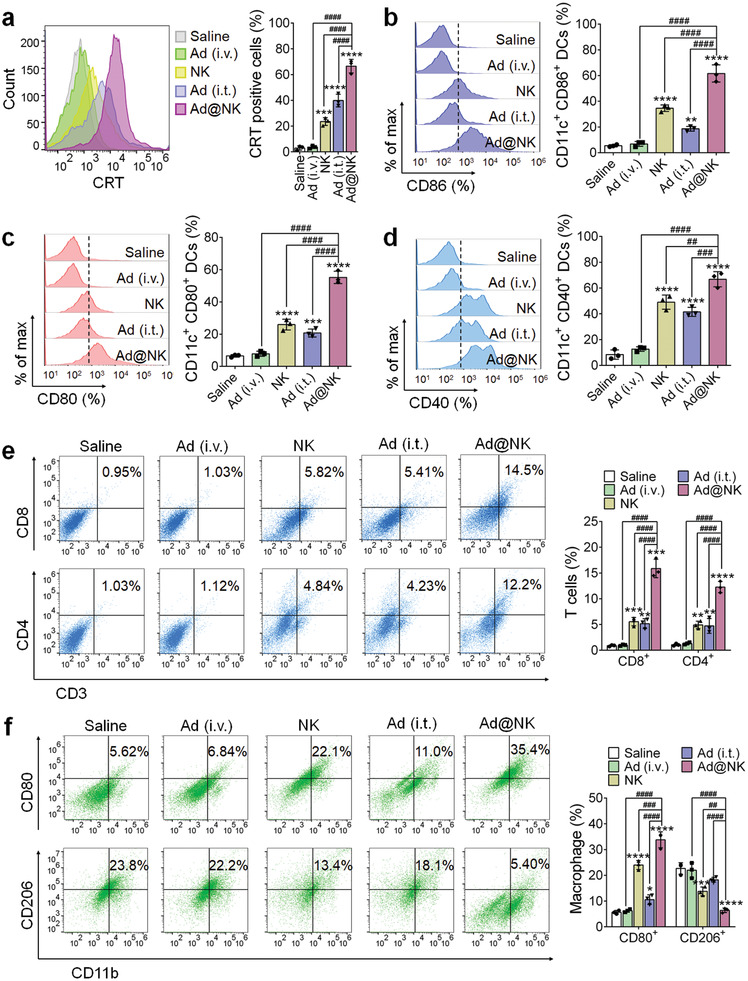
Ad@NK‐mediated immune activation in vivo. a) At day 9 post administration, the primary tumors were harvested from a random part of mice in each group, and then were dissociated into single cell suspension for the immune fluorescent labeling prior to the FACS analysis for the quantification of the cellular rates of CRT^+^. b–d) Cells in the lymph nodes were collected on day 9 for an assessment by flow cytometry after staining with the antibodies against CD11c, CD86, CD80, and CD40. e) Flow cytometric analysis of the intratumor infiltration of CD3^+^CD4^+^ T and CD3^+^CD8^+^ T cells. f) Flow cytometric analysis of CD11b^+^CD80^+^ (M1) and CD11b^+^CD206^+^ (M2) macrophages. Data are represented as mean ± SD, *n* = 3. *P < 0.05; **P < 0.01; ***P < 0.001; ****P < 0.0001 (vs Saline); ^##^ *P* < 0.01; ^###^ *P* < 0.001; ^####^ *P* < 0.0001 (vs indicated) denote significant difference.

In the final set of this study, we focused on elaborating the mechanisms of the combinational antitumor strategy based on Ad@NKs. Multiple evidences suggest that Ad‐induced ICD can recruit and motivate APCs and immunologic effector cells for immune activation.^[^ ^36^ ^]^ To clarify whether Ad@NK treatment would induce a similar effect, we first determined the amount of surface‐exposed CRT, a key indicator for the ICD of cancer cells that is hallmarked by the emission of DAMPs, which has been reported to attract immunological recognition, thus increasing the maturation of dendritic cells (DCs), thereby boosting T cell activity.^[^ ^32^, ^37^ ^]^ As shown in **Figure** **7**a, compared to the saline‐treated group, treatment with Ad@NKs (*P* < 0.0001), Ads (i.t.) (*P* < 0.0001), and NK cells (*P* < 0.001) induced increased CRT positivity rates in the tumors, whereas Ads (i.v.) showed no contribution to the induction of ICD. Moreover, higher maturation rates in DCs of different types (CD11c^+^CD86^+^, CD11c^+^CD80^+^, and CD11c^+^CD40^+^) were also found in the groups treated with Ad@NKs (*P* < 0.0001), Ads (i.t.) (*P* < 0.01) and NK cells (*P* < 0.0001), than those in the saline‐treated control (Figure 7b–d), which further increased the populations of cytotoxic T cells (CD3^+^CD8^+^) (*P* < 0.01) and helper T cells (CD3^+^CD4^+^) (*P* < 0.01) in the three groups (Figure 7e), in agreement with previous reports.^[^ ^38^, ^39^ ^]^ It was noteworthy that Ad@NK treatment led to an increase of 22.0‐, 9.0‐, 17.6‐, and 11.4‐fold in the percentages of CRT^+^ cancer cells (*P* < 0.0001), total mature DCs (*P* < 0.0001), CD8^+^ (*P* < 0.001), and CD4^+^ (*P* < 0.0001) T cells, respectively, in comparison with the saline‐treated control, superior to any other treatments including Ads (i.t. or i.v.) and NK cells (*P* < 0.01), indicating the optimal immunological activity mediated by T lymphocytes (Figure 7a–e).

In addition to the activation of DCs and T cells, we also found that Ad@NK treatment promoted the repolarization of macrophages (Figure 7f), another way for the activation of antitumor immunity, shown by enhanced phagocytosis of cancer cells by proinflammatory M1 macrophages in the TME.^[^ ^40^, ^41^ ^]^ The percentage of M1‐type macrophages (CD11b^+^CD80^+^) in the Ad@NK group increased by 6.0‐fold in comparison with that in saline‐treated control (*P* < 0.0001), higher than the 4.2‐ and 1.9‐fold change in the groups of NK cells (*P* < 0.0001) and Ads (i.t.) (*P* < 0.05) (Figure 7f), respectively, indicating that macrophage repolarization is mainly attributed to the NK cells,^[^ ^42^ ^]^ which can be strengthened by additional Ad treatment. In the meantime, Ad@NK treatment decreased the percentage of M2 macrophages (CD11b^+^CD206^+^) which have an immunosuppressive function,^[^ ^43^ ^]^ the most in comparison to the other treatments (Figure 7f).

To gain better insight into the activation of immune cells, we also quantified blood markers such as granzyme B, IL‐6, IL‐12p40, TNF‐*α*, and IFN‐*γ*, which were reported to be released by activated immunocytes in response to ICD signals.^[^ ^34^, ^40^ ^]^ At 36 h post Ad@NK injection, the amount of granzyme B in the blood increased in comparison to that in NK‐treated mice (*P* < 0.05) (Figure S14a, Supporting Information), indicating that Ad treatment stimulated NK cells to excrete granzyme B in vivo in a STAT4‐dependent manner, which had been shown in our in vitro data (Figure 3 and Figure S9, Supporting Information). In addition, we also found increased blood levels of IL‐6, IL‐12p40, TNF‐*α*, and IFN‐*γ* at day 9 post Ad@NK treatment, in comparison with those in the groups treated with Ads (i.t.) (*P* < 0.0001), NK cells (*P* < 0.0001), Ads (i.v.) (*P* < 0.0001), and saline (*P* < 0.0001) (Figure S14b, Supporting Information). As reported previously,^[^ ^29^, ^34^, ^40^ ^]^ IL‐12p40, TNF‐*α*, and IFN‐*γ* are all powerful cytotoxic factors or cytokines, which are highly correlated with the therapeutic efficacy of Ad@NKs in TNBC mice (Figure 6).

To distinguish the induction of antiviral and antitumor immune response by Ad@NKs, we harvested the serum from the mice treated with Ad@NKs at day 9 to assess the immune serum reaction with uninfected or Ad‐infected 4T1 cells. Compared with negative control serum, the immune serum was specific to 4T1 cells, regardless of Ad infection (Figure S15a,b: Supporting Information). The immunofluorescence was localized only in the cytomembrane of uninfected cells, but in both the surface and interior of infected cells, indicating that the serum antibodies recognized both tumor‐associated surface antigen and intracellular Ads (Figure S15a, Supporting Information). Moreover, the fluorescence intensity in Ad‐infected cells was approximately twice of that in uninfected cells, revealing that the immune response induced by Ad@NKs contributed equally to tumor and virus (Figure S15b, Supporting Information). To further confirm the serum neutralizing effect against virus, we performed neutralizing antibody assay. Compared with negative control serum, the immune serum effectively neutralized the infective activity of Ads in a dose‐dependent manner (Figure S15c, Supporting Information). The increased antiviral immune response also inhibited in vivo replication of Ads, thereby contributing to the slow decrease of intratumor Ad level from day 6 post injection (Figure S11, Supporting Information). Fortunately, the activated antitumor immune response by Ad@NK system overcompensated for the viral reduction, leading to a higher immune efficacy for TNBC than Ads (i.t.) and other regimens (Figure 7 and Figure S14b, Supporting Information).

Since immunotherapy sometimes leads to serious toxic and side effects, ^[^ ^44^ ^]^ we also assessed the safety of Ad@NKs. Compared with untreated control, Ad@NK‐treated mice showed no significant difference in body weights, major hepatorenal function index (ALT activity, AST activity, and BUN concentration), and blood routine and biochemical indexes (WBC, RBC, Hb, platelet, neutrophil, lymphocyte, monocyte, and RBC distribution width) (Figure S16a–c, Supporting Information).^[^ ^45^ ^]^ In addition, the histological data indicated that Ad@NK treatment did not induce remarkable injury in the main organs (Figure S16d, Supporting Information).

In general, these data elucidated the relationship between Ad@NK treatment and in vivo anticancer immunity, indicating that Ad@NKs triggered ICD, thereby recruiting and activating multiple types of immunocytes for an enhanced anticancer immune response. Beyond this, Ad@NK treatment was safe enough to avoid causing toxic and side effects.

## 3 Discussion

Recently, several oncolytic viral drugs have been approved for clinical trials through i.t. treatment.^[^ ^3^ ^]^ One of the most common approaches to construct a tumor‐selective virus is a 24‐bp deletion (Δ24) from the E1A gene to block the E1A‐Rb interaction, thus inhibiting Ad replication in normal cells. In contrast, disruption of the Rb pathway and increased activity of the E2F1 promoter in cancer cells still allowed effective replication of Ads inside cells, despite the mutation in the E1A region,^[^ ^46^ ^]^ thereby leading to a faster replication and shorter infection cycle of Ads in tumor cells than in normal cells (Figures S3 and S4, Supporting Information). Currently, the strategy of MSC‐mediated tumor‐targeted delivery of oncolytic viruses further accelerated the translational process of tumor virotherapy in an intravenous route instead of i.t. by overcoming immunogenic barriers.^[^ ^13^, ^14^ ^]^ However, MSCs can accelerate tumor growth by direct or indirect malignant transformation or suppressing host immune responses.^[^ ^16^, ^17^ ^]^ Moreover, the cancer‐inducible microenvironment in patients increases the pro‐metastatic activity of MSCs through an accelerated EMT.^[^ ^18^ ^]^ To avoid these risks, NK cells were used as a replacement for MSCs in this study. NK cell‐based carriers with a high tumor‐homing tropism took over the advantages of MSCs to act as bioreactors for the loading, replication, and amplification of Ads, as well as serving as shelters for blocking antibody neutralization, thereby conducting a highly efficient targeted delivery of Ads (Figures 1, 2, 5, and 6e and Figure S12, Supporting Information) for a tumor‐selective cytotoxicity (Figures 1a and 4, Figures S2 and S5, Supporting Information). Unlike MSCs, NK cells are strong immunologic effectors, providing an efficient complement to the virotherapy based on Ads for the induction of a strengthened therapeutic efficacy in TNBC (Figures 3, 4, 6 and Figure S2, Supporting Information).

Rather than the effect of “1 + 1 = 2,” systemic treatment of Ad@NKs led to a higher antitumor activity than the combination of Ads (i.v.) and NKs (i.v.), even not weaker than Ads (i.t.) combined with NKs (i.v.) (Figure S13, Supporting Information). On one hand, Ads carried by NK cells provably led to a much more efficient tumor targeting and virotherapy than intravenous treatment of free Ads (Figures 5 and 6). On the other hand, Ad‐loaded NK cells showed a higher immunotoxic effect than unloaded cells, providing a powerful supplement to Ad‐mediated cancer cell killing (Figures 3, 4, 6, and Figure S2, Supporting Information), owing to the activation of the STAT4‐granzyme B‐dependent pathways (Figure 3). As reported recently,^[^ ^47^ ^]^ Ad infection can stimulate the antiviral activity of endogenous NK cells, thereby attracting NK cells to attack infected ovarian cancer cells, although the related molecular mechanisms for this are still unclear. To clarify the regulatory mechanisms involving Ads and NK cells, series of experiments were carried out in this study, which demonstrated that NK cells showed a response to Ad stimulation through increased expression of IFN‐*α* and IFITs, thus activating type I interferon signaling pathways (Figure 3 and Figure S9, Supporting Information).^[^ ^26^ ^]^ Similar to the signal transduction in T lymphocytes,^[^ ^28^ ^]^ activated type I interferon signaling pathways in NK cells also promoted the secretory expression of granzyme B, a cytotoxic molecule that induces cancer cell apoptosis,^[^ ^29^ ^]^ in a STAT4‐dependent manner (Figure 3). Nevertheless, there are some subtle differences in the upregulation of STAT4 in T and NK lymphocytes after type I interferon signaling pathways are activated. In NK cells, the mRNA, total, and phosphorylated (Tyr693) protein levels of STAT4 were all upregulated (Figure 3), whereas only the level of phosphorylated STAT4 protein showed a significant increase in T cells, which actuated the nuclear import of STAT4 for binding upstream of the granzyme B promoter, thus enhancing transcription.^[^ ^28^ ^]^ As indicated by the follow‐up in vitro and in vivo data, the enhanced cytotoxicity activity of NK cells, coupled with their therapeutic cargos, led to a combined antitumor effect of “1 + 1 > 2” on TNBC (Figures 4, 6 and Figures S2, S13, and S14a, Supporting Information), an aggressive breast cancer subtype indicating poor prognosis and high rates of metastasis and relapse in the clinic.^[^ ^48^ ^]^

It has become clear that metastasis is an acknowledged obstacle for the cure of cancer, which is governed not only by the intrinsic mechanisms of cancer cells but also by the TME and systemic factors, indicating that whole‐body, not local, antineoplastic activity is required for defending metastasis.^[^ ^49^ ^]^ The constant improvement of immunotherapies presents a potential solution for cancer metastasis by waking up the sleeping immune system, inducing a systemic immune response against cancer, and thus significantly increasing the survival of patients with metastasis.^[^ ^50^ ^]^ As mentioned in numerous studies,^[^ ^49^, ^51^ ^]^ NK cells monitor and suppress the metastatic dissemination of cancer cells. Moreover, the antitumor inflammatory and immune responses can also be facilitated by the treatment of oncolytic Ads or NK cytomembranes, depending on the activation of cytotoxic T lymphocytes (CTLs), which are commonly boosted by DC‐ and macrophage‐mediated antigen presentation.^[^ ^41^, ^42^, ^52^, ^53^ ^]^ The key indicators for the activation of NK cells and CTLs, such as increases in levels of granzyme B, IL‐6, IL‐12p40, TNF‐*α*, IFN‐*γ*, ICD‐positive cancer cells, mature DCs, M1‐type macrophages, CD4^+^, and CD8^+^ T cells,^[^ ^32^, ^34^, ^39^, ^40^, ^42^, ^54^ ^]^ were all observed in mice treated with Ad@NKs, in comparison to those in other treatment groups (Figure 7 and Figure S14, Supporting Information). The stimulation of immune responses was not only due to the contribution of living NK cells or Ads, but also attributed to the cytomembranes of dead NK cells after releasing their cargos,^[^ ^10^, ^42^, ^53^ ^]^ consequently leading to the growth inhibition and apoptosis of cancer cells that were no longer confined to the primary tumors, but also worked in the whole body to prevent the genesis and development of metastatic lesions (Figures 6, 7, and Figure S14, Supporting Information). It is worth noting that the systemic immune activation did not induced significant toxic and side effects, revealing a high‐level safety of Ad@NKs as an anticancer agent (Figure S16, Supporting Information).

Until now, both engineered oncolytic viruses and NK cells have entered clinical trials, showing no significant adverse effects in patients,^[^ ^3^, ^55^ ^]^ and efficient antineoplastic activity.^[^ ^5^, ^56^ ^]^ Their favorable safety and promising anticancer efficacy offer encouraging prospects for oncolytic viruses and NK cells, urging us to speed up translational research on biohybrid Ad@NK system, which presented encouraging curative efficacy and high safety in mice bearing highly metastatic TNBC xenografts. In addition to TNBC, the therapeutic functions of Ad@NKs can be investigated in other types of cancers for a more comprehensive evaluation of the anticancer spectrum. To better understand the cell behaviors of NK cells in response to Ad infection, a series of studies on the levels of transcription, translation, and post‐translational modification can also be performed in future studies to expound the interplay between Ads and NK cells. Moreover, we are also working on the genetic, biological, structural, and chemical engineering of NK cells for a higher amplification efficiency of Ads and a prolonged survival time after Ad infection. Above all, the pharmacodynamic and pharmacokinetic properties and side effects of Ad@NKs should be tested in different animal models beyond mice to eliminate the potential safety hazards in future clinical studies.

## 4 Conclusion

In conclusion, we have demonstrated that a biohybrid NK cell‐based Ad delivery system possesses excellent functions of growth inhibition, apoptosis promotion, and immune activation against the development and metastasis of TNBC in mice. The internal environment of NK cells is suitable for the loading, replication, and amplification of Ads, thereby leading to an efficient targeted delivery and a logarithmically increased replication in tumor tissues, by blocking the interaction between Ads and neutralizing antibodies in the systemic circulation to inhibit immune clearance. In return, Ad augments the anticancer immunity of NK cells by upregulating the type I interferon signaling pathways in a STAT4‐ and granzyme B‐dependent manner. The complementary interplay between Ads and NK cells in combinational immunotherapy and virotherapy is a promising therapeutic strategy in oncology, which has promising clinical applications in the future.

## 5 Experimental Section

### Ethics Statement

All in vivo experiments were approved by the Institutional Animal Care and Use Committee of Yangzhou University and performed in compliance with the guidelines of Jiangsu Laboratory Animal Welfare and Ethical of Jiangsu Administrative Committee of Laboratory Animals.

### Key Reagents

All the key reagents used in this study were shown in Table S1 (Supporting Information). The primers used for qRT‐PCR in this study were synthesized by TSINGKE Biological Technology Co., Ltd (Beijing, China) and their sequences were listed in Table S2 (Supporting Information).

### Cell Culture

4T1 mouse TNBC cells, MDA‐MB‐231 human TNBC cells, L929 mouse fibroblasts, Nrk‐52e rat renal proximal tubular cells, mouse NK cells, and 293A cells were provided by Cell Bank, Chinese Academy of Sciences (Shanghai, China). The 4T1, MDA‐MB‐231, L929, and Nrk‐52e cells were cultured in DMEM medium, supplemented with 10% fetal bovine serum, 100 U mL^−1^ penicillin, and 100 mg mL^−1^ streptomycin. In addition to the culture system for 4T1 and others, 0.1 × 10^−3^ m NEAA and 2 × 10^−3^ m l‐glutamine were added to the culture medium for 293A cells. The NK cells were suspension‐cultured in alpha‐MEM, supplemented with 12.5% fetal bovine serum, 12.5% horse serum, 2 × 10^−3^ m l‐glutamine, 1.5 g L^−1^ sodium bicarbonate, 0.2 × 10^−3^ m inositol, 0.1 × 10^−3^ m 2‐mercaptoethanol, 0.02 × 10^−3^ m folic acid, and 100 U mL^−1^ IL‐2.

### Ads

The tumor‐selective Ads applied in this study was packaged, purified and tittered by Fubio Biotechnology Co., Ltd (Suzhou, China). In brief, the shuttle plasmid that carries an EGFP reporter gene and a mutant E1A coding gene containing a 24‐bp deletion in the region responsible for binding Rb protein,^[^ ^22^ ^]^ along with a helper adenovirus plasmid of pBHG lox ΔE1,3 Cre,^[^ ^57^ ^]^ were co‐transfected into 293A cells for the packaging of Ads.

### Determination of Ad Infectivity Titer

To make a normalized assessment of Ad infectivity in different types of cells, the PFU was quantified by plaque assay in a reference cell line 293A.^[^ ^22^ ^]^

### Determination of Ad Copy Number

Ad genome was extracted from medium, serum, blood, or tissue lysate using a mammalian genomic DNA extraction kit. The Ad copies were quantitated by qRT‐PCR analysis of EGFP gene. A plasmid coding EGFP was used as the standard template.

### Co‐Incubation of Ads and NK Cells

To acquire effective Ad loading, hexadimethrine bromide (8 µg mL^−1^) that acts as a promotor for virus infection was added into the mixture containing NK cells and Ads at different MOIs for the co‐incubation throughout this study.^[^ ^58^ ^]^

### Preparation of Ad@NK

Ad@NK was prepared by the co‐incubation of NK cells and Ads at MOI 800 for 48 h. Upon the ending of incubation, the cells were harvested for twice PBS washing prior to the subsequent studies.

### Cytotoxicity Induced by Ads in NK Cells

The CCK‐8 assay was performed to evaluate Ad‐induced cytotoxicity in NK cells. In brief, NK cells were seeded into a 96‐well plate and co‐incubated with Ads at MOI 400 or 800 for 24, 48, 72, 96, and 120 h, respectively. Afterward, WST‐8 solution was added into the wells. 1 h later, OD_450_ of each well was measured to evaluate the cell viability.

### NK Cell Uptake of Ads

Ads were fluorescent labeled by a co‐incubation of Cy5‐NHS (EX/EM: 646/664 nm), then were added to NK cells. After the intervals of 0.5, 1, 3, 6, and 12 h, respectively, the Cy5 fluorescence in the cells was quantitated by FACS and photographed under a laser confocal microscope.

### Dot Blot

Ads (4 × 10^6^ PFU), NK cells (2 × 10^5^ cells), and Ad@NKs (2 × 10^5^ cells loading with 4 × 10^6^ PFU of Ads) treated with PBS or RIPA cell lysis buffer were spotted onto a nitrocellulose membrane, followed by a BSA blocking and an incubation with mouse anti‐Adenovirus hexon protein and HRP‐conjugated anti‐mouse IgG in turn. The targeted spots were detected in an ECL chemiluminescence system.

### Ad‐Loading Efficiency of NK Cells

After the treatments of Ads at MOI 400 or 800 for 24, 48, 72, 96, and 120 h, respectively, NK cells were harvested for assessing the intensity of EGFP fluorescence by FACS and fluorescence microscope. Moreover, Ads in the cells were extracted by freeze‐thaw, the titer of which was determined in 293A cells.

### Ad Release from NK Cells

Ad@NKs were seeded into fresh medium for the incubation time of 24, 48, 72, and 96 h, respectively. After removing the cells by centrifugation, the medium containing released Ads were collected, and then were tittered in 293A cells.

### Infection Cycles of Ads in NK and 4T1 Cells

Ads (MOI 800) were added to NK cells or 4T1 cells for an incubation without hexadimethrine bromide. 2 h later, the medium containing Ads was replaced by a fresh one. At the intervals of 0, 6, 12, 18, 24, 48, 72, and 96 h, the medium was harvested for a determination of Ad copy number. In the meanwhile, the cells were constantly cultured in a fresh medium. At the end of the first infection cycle, Ad genome began to be detectable in the medium.

### Ad Infection in Normal and Tumor Cells

At the intervals of 0, 24, 48, and 72 h after treated with Ads at MOI 5 or 10, 4T1, MDA‐MB‐231, L929, and Nrk‐52e cells were photographed under a fluorescence microscope to assess the infectivity of Ads.

### Cytotoxicity in 4T1 Cells

2 × 10^3^ 4T1 cells were seeded into 96‐well plates and cultured overnight prior to the treatments of Ads (MOI 0, 0.3125, 0.625, 1.25, 2.5, 5, or 10), NK cells (E: T/NK: 4T1 = 0, 0.015625, 0.03125, 0.0625, 0.125, 0.25, or 0.5) or Ad@NKs (MOI 0–10; E: *T* = 0–0.5) for 48, 72, 96, and 120 h, respectively. Upon the treatments ended, the suspension‐cultured NK cells were washed out with PBS, followed by the treatment of WST‐8 for the performance of CCK8 assay to evaluate the cell viability of 4T1. The CI value was calculated according to the Chou‐Talalay equation to evaluate the synergistic effect of Ads and NK cells.^[^ ^30^ ^]^

### Cytotoxicity in Tumor and Normal Cells

2 × 10^3^ MDA‐MB‐231, L929, and Nrk‐52e cells were seeded into 96‐well plates and cultured overnight prior to the treatment of Ads (MOI 10) for 48, 72, 96, and 120 h, respectively. WST‐8 solution was subsequently added into the wells for measuring the cell viability.

### Uptake of Ads by Tumor Cells

Ads were fluorescent labeled by a co‐incubation of Cy5‐NHS (EX/EM: 646/664 nm), then were added to 4T1 and MDA‐MB‐231 cells. After the intervals of 1, 3, and 6 h, respectively, the Cy5 fluorescence in the cells was quantitated by FACS analysis.

### Apoptosis in 4T1 Cells

At day 2 post the seeding of 10^4^ 4T1 cells in 24‐well plates, Ads (MOI 10), NK cells (E: T = 0.5) or Ad@NKs (MOI 10, E: T = 0.5) were added to the cells for a 72‐h incubation. Subsequently, the wells were washed with PBS to remove suspended NK cells, followed by fixing in 4% paraformaldehyde and TUNEL staining, which was performed per the kit manual to record the apoptotic cells under a fluorescence microscope.

### ICD of 4T1 In Vitro

Having undergone the treatments described above, 4T1 cells were harvested and incubated with an antibody targeting CRT for FACS analysis to assess the percentages of the cells showing positive CRT‐exposure. After removing the cells by centrifugation, the amounts of extracellular released HMGB1 and ATP were quantified in the medium by ELISA and ATP assays, respectively. ELISA assay was performed by Servicebio Biotechnology Co., Ltd (Wuhan, China)

### Molecular Regulation Mechanism in NK Cells in Response to Ad Treatment

Untreated and Ad‐treated (MOI 800, 48 h) NK cells were harvested and sent to CapitalBio Technology Co., Ltd (Beijing, China) for a whole transcriptome sequencing. Moreover, the abundance levels of the DEGs and the related immunological regulator genes in the cells were further evaluated by qRT‐PCR, western blot, and ELISA. The ELISA assay was carried out by Servicebio Biotechnology Co., Ltd (Wuhan, China).

### In Vivo Delivery of Ads

10^5^ 4T1 cells were injected into the fat pad of lower left breast of 4‐week‐old female BALB/c mice with a body weight of 18–22 g, which were obtained from Laboratory Animal Centre of Yangzhou University. When the tumors grew up to a size of ≈80 mm^3^, PBS, Cy5‐Ads, DiR‐NK cells (NK cells labeled with DiR iodide, EX/EM: 754/778 nm), and Cy5‐Ad@DiR‐NKs (DiR‐NK cells previously co‐incubated with Cy5‐Ads at MOI 800 for 48 h) were injected into the caudal veins of tumor‐bearing mice (*n* = 3), respectively. After the intervals of 0.5, 6, 24, and 48 h, the in vivo fluorescence imaging was performed by using an IVIS Lumina imaging system and the amount of DiR and Cy5 in tissues was analyzed using IVIS Living imaging software. The tumor tissues and main organs were harvested from the sacrificed mice at 48 h post‐administration for an additional fluorescent quantitation. Furthermore, another group of tumor‐bearing mice injected with Ad@NKs were sacrificed at day 4, 6, 8, 10, 12, and 14 post injection for a quantitation of intratumor Ad copies (*n* = 3), respectively.

### In Vivo Ad Release from NK Cells

Ad@NKs were administered to tumor‐free mice intravenously (*n* = 3). After 6, 12, 24, 36, 48, and 72 h, the viral concentration in serum was determined by qRT‐PCR analysis, respectively.

### Pharmacokinetics of Ads Carried by NK cells

Free Ads or Ad@NKs were administered to tumor‐free mice intravenously (*n* = 3). After 0.125, 0.5, 1, 2, 4, 8, 12, 24, 48, 72 h, the viral concentration in blood was determined by qRT‐PCR analysis, respectively. Based on these data, the half‐life (t_1/2_) was calculated.

### In Vivo Anticancer Efficacy

Orthotopic 4T1 breast tumor model was established as above. When the tumors grew up to a size of ≈80 mm^3^, mice were randomly divided into 5 groups for different treatments of saline (i.v.), Ads (4 × 10^7^ PFU per mouse, i.t.), Ads (4 × 10^7^ PFU per mouse, i.v.), NK cells (2 × 10^6^ cells per mouse, i.v.), and Ad@NKs (2 × 10^6^ NK cells carrying 4 × 10^7^ PFU of Ads per mouse, i.v.). The primary tumor size was measured every two days until the end of a 14‐d treatment course (*n* = 6). In addition, the tumor size was also measured to compare the tumor growth rate in the groups treated with Ads (i.v.) + NKs (i.v.), Ads (i.t.) + NKs (i.v.), and Ad@NKs (*n* = 5). 36 h later, the mice treated with NK and Ad@NK, respectively, were sacrificed for the quantification of granzyme B in blood by ELISA (*n* = 3). At day 9 post administration, the blood, primary tumors, and lymph nodes were harvested from a random part of mice in each group. Afterward, the tumors were dissociated into single cell suspension by using DMEM containing 0.2% collagenase, 0.01% hyaluronidase, and 0.002% DNase I. One the one hand, the cells were subjected to FACS analysis after the incubation with the antibodies against the surface markers of CRT, CD3, CD4, CD8, CD11b, CD80, and CD206, respectively or combined (*n* = 3). On the other side, Ads were extracted from the digested tumor cells in the groups of Ads (i.v.), Ads (i.t.) and Ad@NKs by repeated freezing and thawing, the titer of which was evaluated in 293A cells to make a quantification analysis for the intratumor infective units (*n* = 3). To evaluate the maturation levels of different types of DCs, the lymph nodes were also digested into single cell suspension for FACS analysis after the incubation with the antibodies recognizing CD11c, CD40, CD80, and CD86. In addition, the amounts of IL‐6, IL‐12p40, TNF‐*α*, and IFN‐*γ* in blood were determined by ELISA (*n* = 3). At day 14 post administration, the primary tumors were harvested from all the rest mice in each group (*n* = 6), in turn, were weighed, photographed, and sectioned for H&E staining and TUNEL assay. Moreover, to make an assessment of pulmonary metastasis, the lung tissues were also harvested from the mice (*n* = 6), all of which were photographed and sectioned for H&E staining analysis after their surface metastases were counted under a dissecting microscope. All of the ELISA and histological studies were conducted by Servicebio Biotechnology Co., Ltd (Wuhan, China).

### Reaction of Immune Serum against Tumor and Virus

Orthotopic 4T1 breast tumor model was established as above. When the tumors grew up to a size of ≈80 mm^3^, Ad@NKs (2 × 10^6^ NK cells carrying 4 × 10^7^ PFU of Ads per mouse) were injected to the mice intravenously. 9 days later, the serum was harvested and then used for immunofluorescence (IF) assay. After BSA blocking, uninfected and Ad‐infected 4T1 cells were incubated with the immune serum and a Cy3‐labeled secondary antibody in turn. The serum from untreated tumor‐free mice was used as a negative control. The fluorescence was observed under a laser confocal microscope.

### Neutralizing Antibody Assay

The serums were prepared as above to assess their neutralizing effect against virus. After a preincubation with different serums in different concentrations at 37 °C for 1 h, Ads (MOI 10) were added to 4T1 cells. 48 h later, the infection rate of 4T1 cells was determined by measuring cellular EGFP intensity. To facilitate comparison, the infection rate by the Ads preincubated with blank serum (1: 10 000) was set to 100%. The serum neutralizing activity was represented by the reduction of infection rate.^[^ ^59^ ^]^

### In Vivo Safety of Ad@NKs

Tumor‐free mice were untreated or treated with Ad@NKs (2 × 10^6^ NK cells carrying 4 × 10^7^ PFU of Ads per mouse, i.v.). The body weight was measured every two days until the end of a 14‐d treatment course (*n* = 3). 72 h post administration, the blood was collected for a determination of hepatorenal function and blood routine and biochemical indexes. At day 14, the main organs (heart, liver, spleen, lung, and kidneys) were harvested from sacrificed mice for H&E staining analysis.

### Statistical Analysis

GraphPad Prism software was used for statistical analyses and to prepare the graphs. Figure legends indicate the sample size in each panel. For the comparison of two groups, a student's *t* test was used. For more than two groups, a one‐way ANOVA was used. *P < 0.05, **P < 0.01, ***P < 0.001, ****P < 0.0001, ^#^ *P* < 0.05, ^##^ *P* < 0.01, ^###^ *P* < 0.001, and ^####^ *P* < 0.0001. Results are shown as mean ± SD.

## Conflict of Interest

The authors declare no conflict of interest.

## Supporting information

Supporting Information

## Data Availability

Research data are not shared.
