# Supplementary material for: An Inter‐Supplementary Biohybrid System Based on Natural Killer Cells for the Combinational Immunotherapy and Virotherapy of Cancer
Source: Adv Sci (Weinh). 2021 Nov 7;9(2):2103470. doi: 10.1002/advs.202103470 (PMC8805568; doi:10.1002/advs.202103470)
Supplement: Supplementary file 1 — Supporting Information [file ADVS-9-2103470-s001.pdf]

© 2021 Wiley-VCH GmbH

ADVANCED SCIENCE
Open Access

Supporting Information

for *Adv. Sci.,* DOI: 10.1002/advs.202103470

An Inter-Supplementary Biohybrid System Based on Natural Killer Cells for the Combinational Immunotherapy and Virotherapy of Cancer

Li Ding, Qingqing Gao, Zhuobin Xu, Liangliang Cai, Sujuan Chen, Xinyue Zhang, Peng Cao, and Gang Chen**

Supporting Information

An Inter-Supplementary Biohybrid System Based on Natural Killer Cells for the Combinational Immunotherapy and Virotherapy of Cancer

Li Ding, Qingqing Gao, Zhuobin Xu, Liangliang Cai, Sujuan Chen, Xinyue Zhang, Peng Cao, and Gang Chen**

Table of contents

Figure S1. Flow diagram for the construction and production of Ads.

Figure S2. Cytotoxicity induced by Ads, NK cells and Ad@NKs in 4T1 tumor cells. Related to Figure 4a.

Figure S3. Ads show a higher infective ability and a faster replication rate in tumor cells than in normal cells.

Figure S4. Infection cycle of Ads was shorter in 4T1 tumor cells than in normal NK cells.

Figure S5. Ads show a higher cytotoxicity in tumor cells than in normal cells.

Figure S6. Human and mouse coxsackievirus and adenovirus receptors have a high sequence similarity.

Figure S7. Mouse cancer cell line 4T1 and human cancer cell line MDA-MB-231 show a similar uptake rate of Ads.

Figure S8. Cellular uptake of Ads by NK cells. Related to Figure 1c-1e.

Figure S9. Transcriptome analysis for the change of mRNA expression profiles in NK cells after Ad treatment. Related to Figure 3.

Figure S10. Background fluorescence in mice under the detectable waveband of DiR and Cy5. Related to Figure 5.

Figure S11. Intratumor replication of Ads in the mice treated with Ad@NKs.

Figure S12. Drug release and pharmacokinetics kinetics of Ads carried by NK cells.

Figure S13. Comparison of Ad+NK treatment effect via different administration schedules in 4T1-bearing mice.

Figure S14. Ad@NK treatment leads to an increased release of cytokines in 4T1-bearing mice.

Figure S15. Ad@NKs induce immune responses against tumor and virus in 4T1-bearing mice.

Figure S16. Safety assessment of Ad@NKs.

Table S1. Key reagents used in this study.

Table S2. Primers used for qRT-PCR.

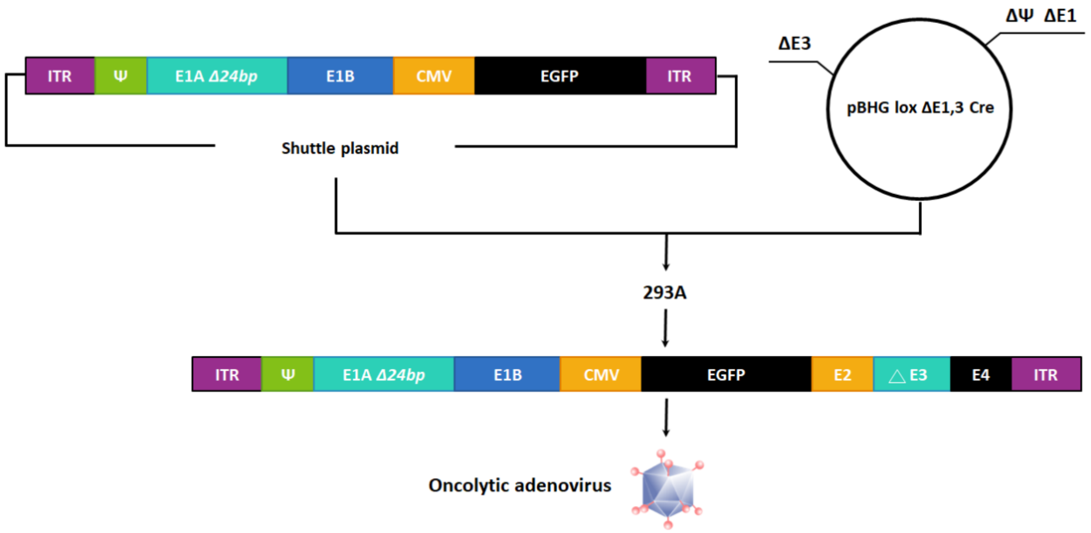

Figure S1. Flow diagram for the construction and production of Ads. A shuttle plasmid carrying an EGFP reporter gene and a mutant E1A coding gene with a 24-bp deletion for a blocked binding to Rb protein, along with a helper adenovirus plasmid of pBHG lox ΔE1,3 Cre, were co-transfected into 293A cells for the packaging of Ads.

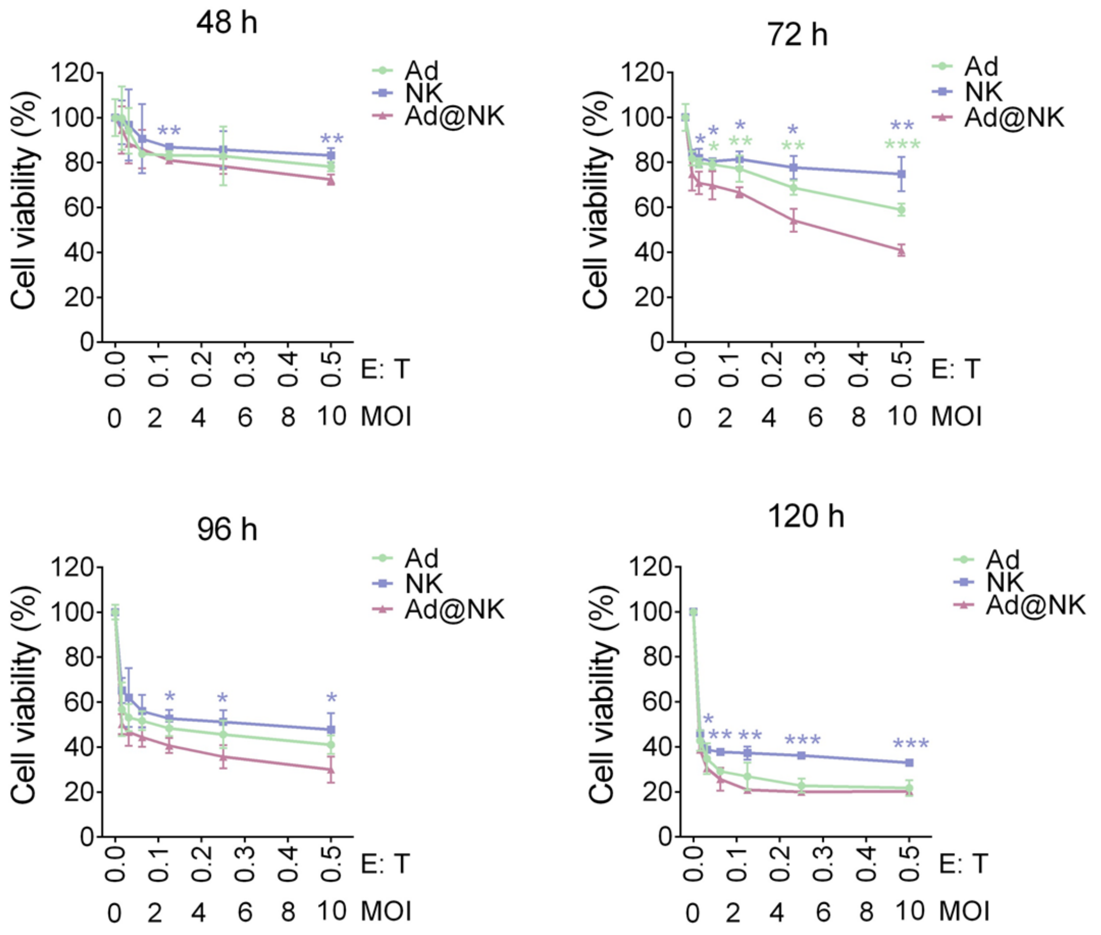

Figure S2. Cytotoxicity induced by Ads, NK cells and Ad@NKs in 4T1 tumor cells. Related to Figure 4a. CCK8 assay was performed to assess the cytotoxicity in 4T1 cells induced by the treatments of Ads (MOI 0, 0.3125, 0.625, 1.25, 2.5, 5 or 10), NK cells (E: T/NK: 4T1 = 0, 0.015625, 0.03125, 0.0625, 0.125, 0.25 or 0.5) or Ad@NKs (MOI 0-10; E: T = 0-0.5) for 48, 72, 96 and 120 h, respectively. Data are represented as mean ± SD, n = 3. $^{*}P < 0.05$; $^{**}P < 0.01$; $^{***}P < 0.001$, denote significant difference.

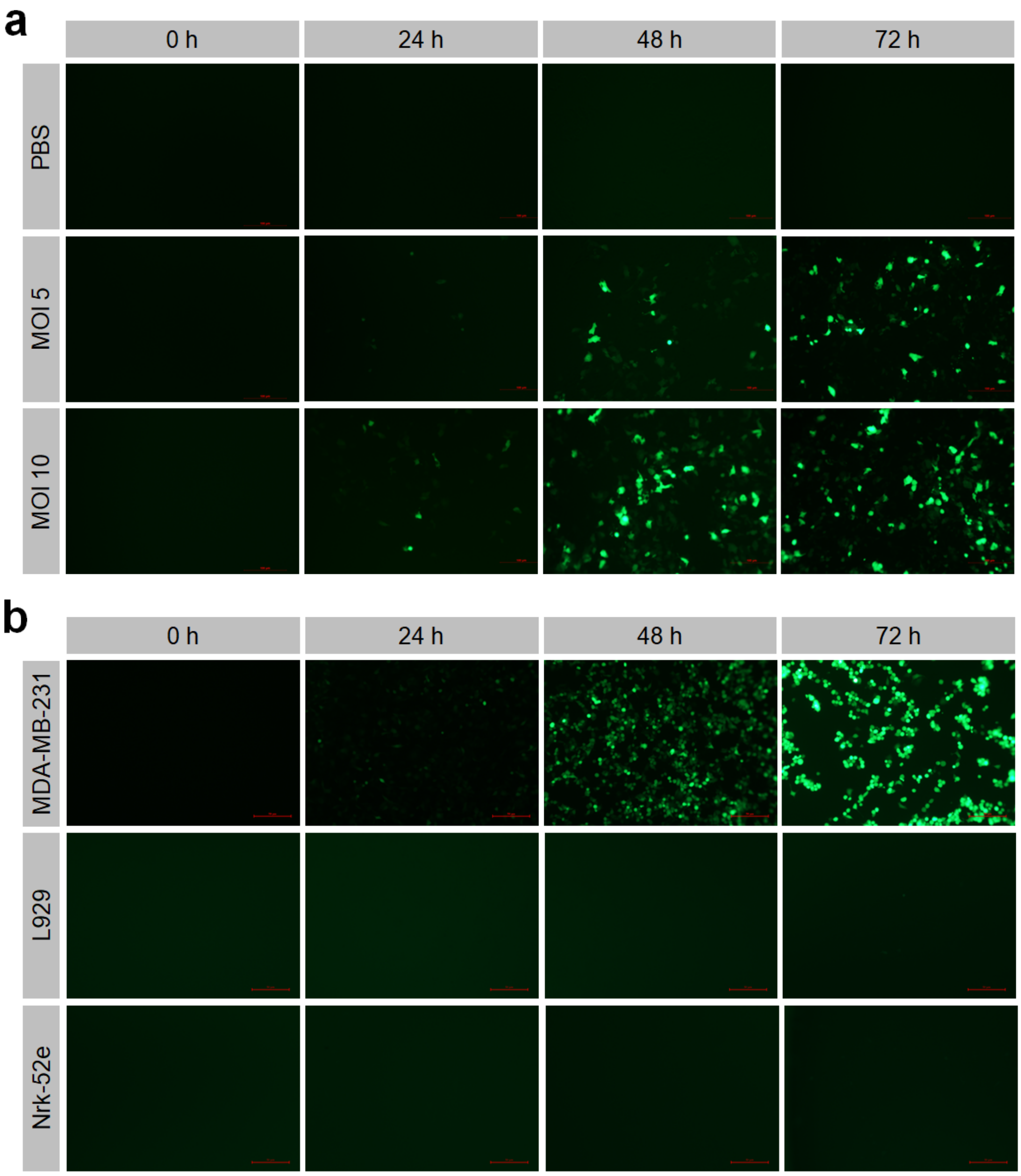

Figure S3. Ads show a higher infective ability and a faster replication rate in tumor cells than in normal cells. a) After treated with PBS or Ads (MOI 5 or 10), the EGFP fluorescence in 4T1 cells was recorded by fluorescence microscope at the time points of 0, 24, 48 and 72 h. Scale bar: 100 μm. b) After treated with Ads (MOI 10), the EGFP fluorescence in MDA-MB-231, L929, and Nrk-52e cells was recorded by fluorescence microscope at the time points of 0, 24, 48 and 72 h. Scale bar: 50 μm.

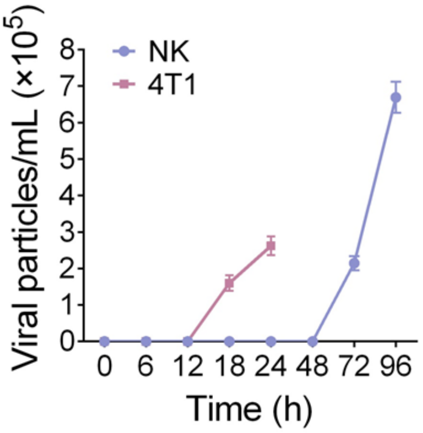

Figure S4. Infection cycle of Ads was shorter in 4T1 tumor cells than in normal NK cells. 4T1 and NK cells were incubated with Ads (MOI 800) for 2 h, followed by a removement of Ads from the medium. Exchange of the cell medium was performed at indicated intervals. The number of adenoviral particles in the replaced medium was determined by qRT-PCR. The first infection cycle of Ads ended when Ad genome began to be detectable. Data are represented as mean ± SD, n = 3.

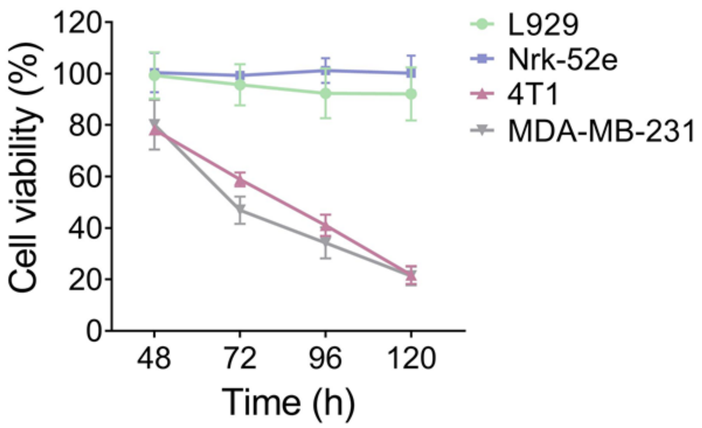

Figure S5. Ads show a higher cytotoxicity in tumor cells than in normal cells. CCK8 assay was performed to assess the cytotoxicity in 4T1, MDA-MB-231, L929, and Nrk-52e cells by the treatment of Ads (MOI 10) for 48, 72, 96 and 120 h, respectively. Data are represented as mean ± SD, n = 3.

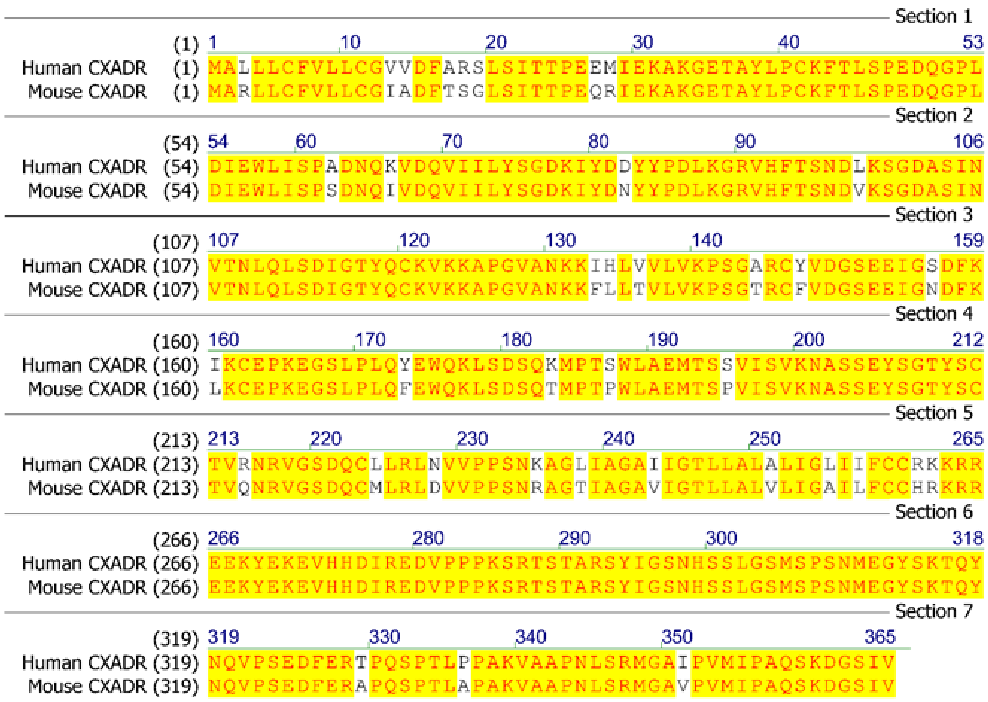

Figure S6. Human and mouse coxsackievirus and adenovirus receptors have a high sequence similarity. The amino acid sequence of mouse CXADR (NM_001025192.3) was compared with that of human CXADR (NM_001338.5) to assess the similarity. The uniform amino acids were highlighted yellow.

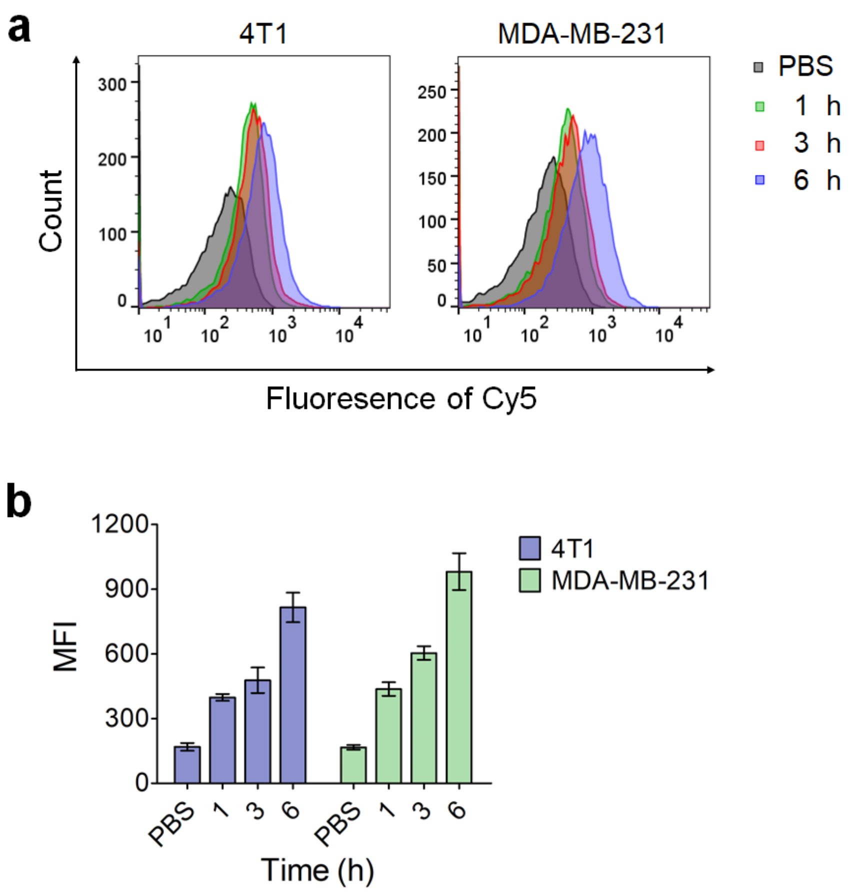

Figure S7. Mouse cancer cell line 4T1 and human cancer cell line MDA-MB-231 show a similar uptake rate of Ads. After incubated with Cy5-Ads for 1, 3, and 6 h, the cell uptake of Ads was evaluated by measuring a) fluorescence peak migration and b) MFI. PBS-treated cells were used as a negative control. Data are represented as mean ± SD, n = 3.

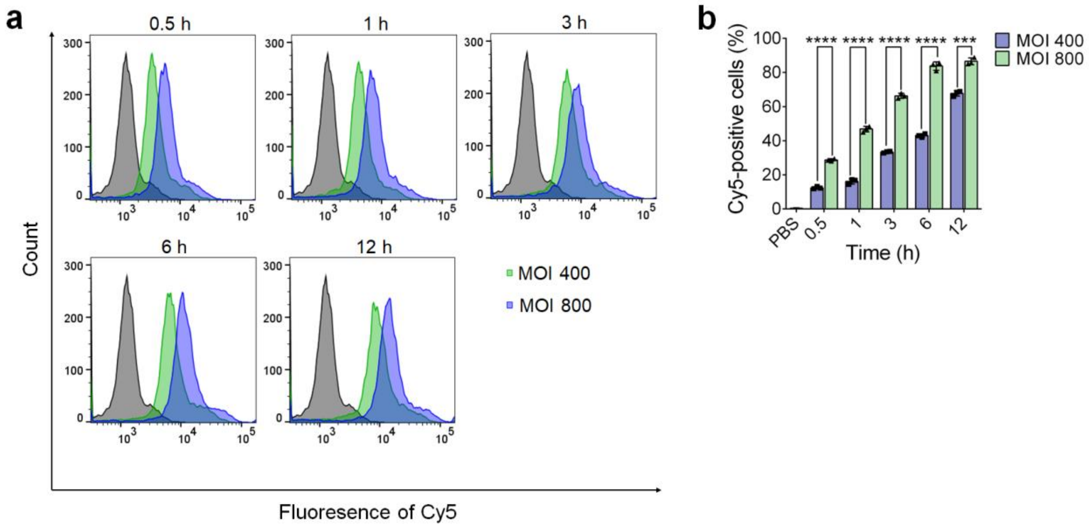

Figure S8. Cellular uptake of Ads by NK cells. Related to Figure 1c-1e. After incubated with Cy5-Ads at different MOI, the NK cell uptake of Ads was evaluated by measuring a) the cellular fluorescence intensity and b) the cellular positive rate of Cy5. Data are represented as mean ± SD, n = 3. $^{***}P < 0.001$; $^{****}P < 0.0001$ denote significant difference.

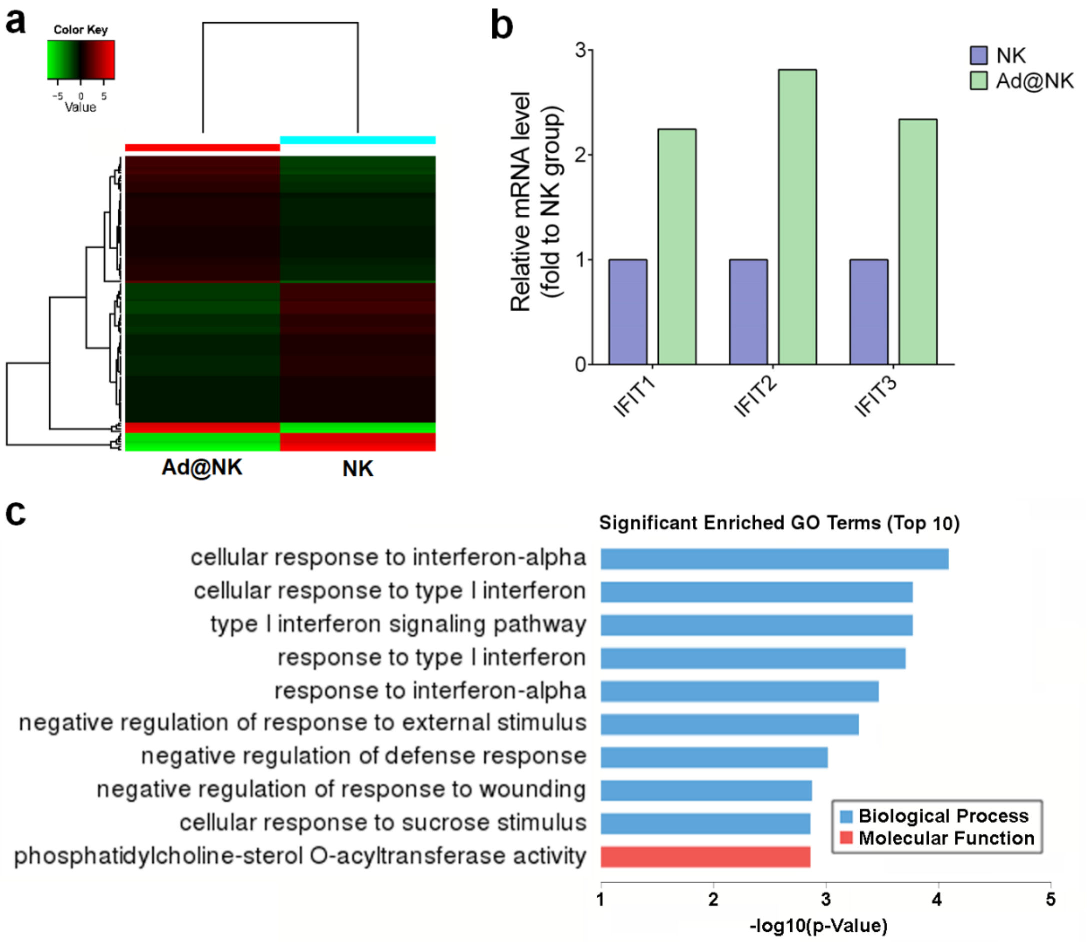

Figure S9. Transcriptome analysis for the change of mRNA expression profiles in NK cells after Ad treatment. Related to Figure 3. After treated with Ads at MOI 800 for 48 h, the NK cells coupled with untreated control were subjected for transcriptome sequencing. a) The differential transcriptional profiles in the two groups were visualized in a cluster heat map. b) DEGs related to immunoregulation in Ad-treated NK cells vs. in the untreated ones. c) Gene Ontology (GO) enrichment analysis indicates the top 10 pathways, in which the DEGs were enriched.

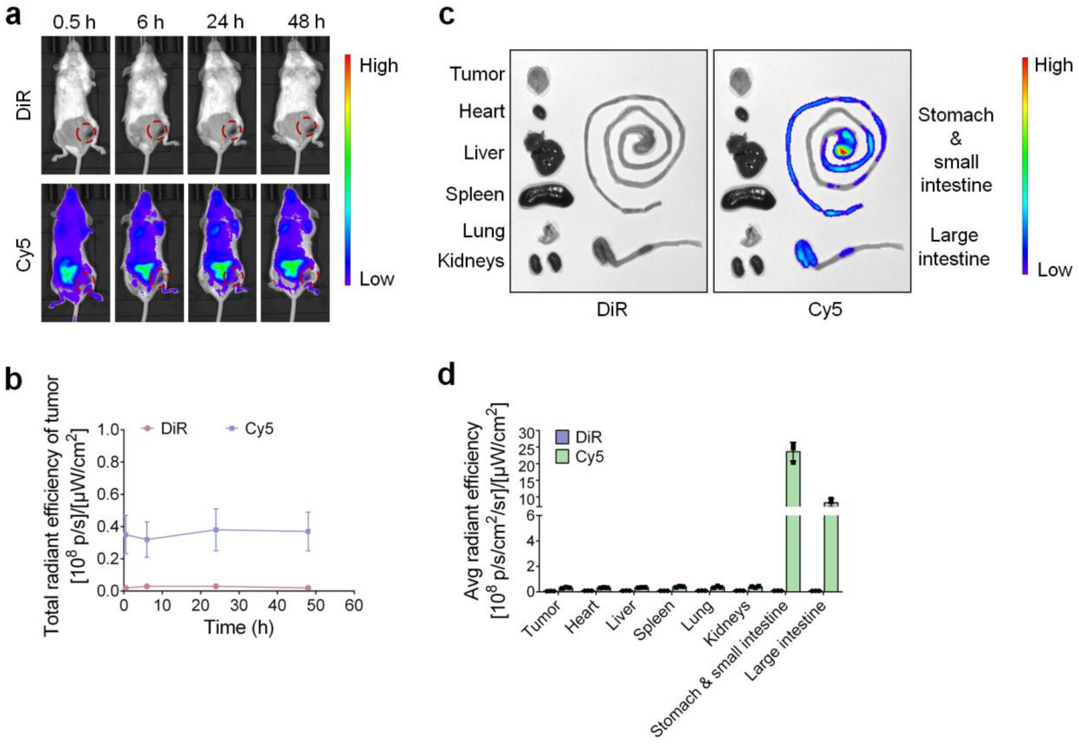

Figure S10. Background fluorescence in mice under the detectable waveband of DiR and Cy5. Related to Figure 5. a) Background fluorescence in the whole body of PBS-treated mice. b) The total radiant efficiencies of tumors were recorded and analyzed using IVIS Living imaging software. c) Fluorescent images were taken for the tumor, heart, liver, spleen, lung, kidneys, stomach, small intestine, and large intestine, which were harvested from the sacrificed mice at 48 h. d) The average radiant efficiencies of different organs were recorded and analyzed using IVIS Living imaging software. Data are represented as mean ± SD, n = 3.

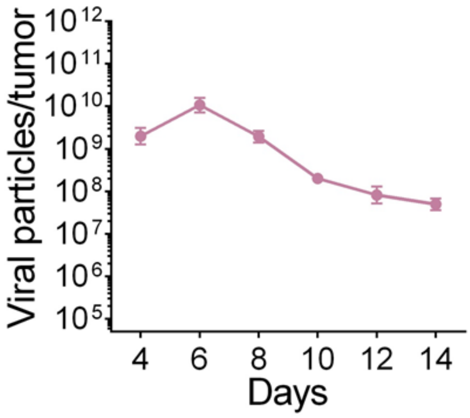

Figure S11. Intratumor replication of Ads in the mice treated with Ad@NKs. At different intervals post intravenous injection of Ad@NKs, the number of intratumor Ad copies was determined by qRT-PCR analysis. Data are represented as mean ± SD, n = 3.

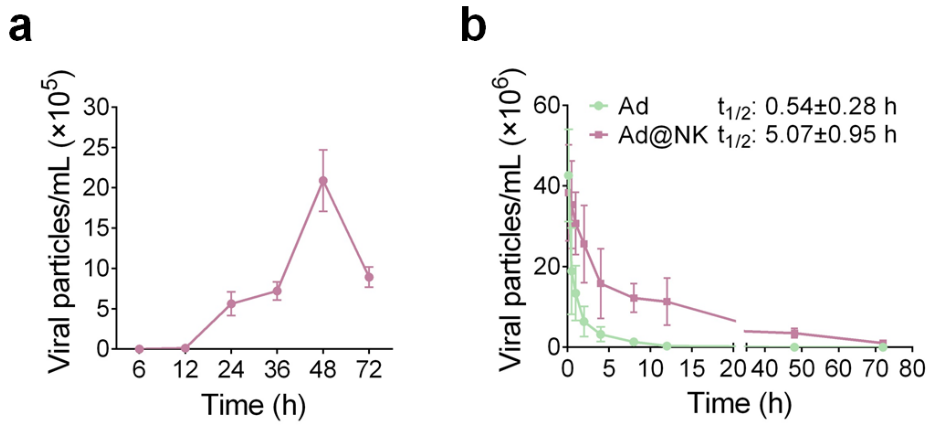

Figure S12. Drug release and pharmacokinetics kinetics of Ads carried by NK cells. a) At indicated intervals post injection with Ad@NKs, the viral concentration in the serum of tumor-free mice was determined by qRT-PCR analysis to study the drug release kinetics of Ads *in vivo*. b) At indicated intervals post injection with Ad@NKs, the viral concentration in the whole blood of tumor-free mice was determined by qRT-PCR analysis to calculate the half-life ($t_{1/2}$) of Ads. Data are represented as mean ± SD, n = 3.

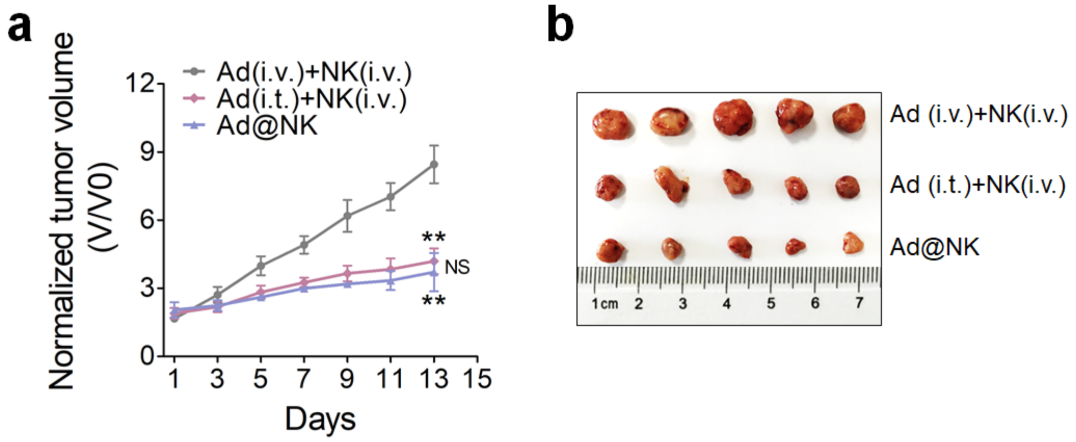

Figure S13. Comparison of Ad+NK treatment effect via different administration schedules in 4T1-bearing mice. a) Tumor growth curves in each group during a 14-day treatment course (n = 5). b) Images of tumors from sacrificed mice at day 14. Data are represented as mean ± SD. $^{**}P < 0.01$ (vs. Ad (i.v.) + NK (i.v.)) denotes significant difference. NS denotes no significant difference between Ad (i.t.) + NK (i.v.) and Ad@NK.

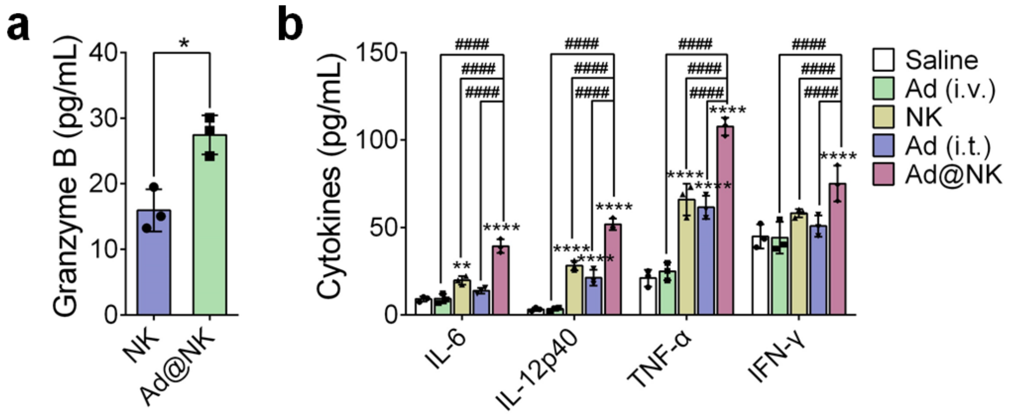

Figure S14. Ad@NK treatment leads to an increased release of cytokines in 4T1-bearing mice. a) Amounts of granzyme B in the blood that collected from the mice at 36 h post NK cell or Ad@NK injection. b) Amounts of IL-6, IL-12p40, TNF-α and IFN-γ in the blood from different treatment groups at day 9. Data are represented as mean ± SD, n = 3. $^{*}P < 0.05$; $^{**}P < 0.01$; $^{****}P < 0.0001$ (vs. indicated or Saline); $^{\#\#\#\#}P < 0.001$ (vs. indicated) denote significant difference.

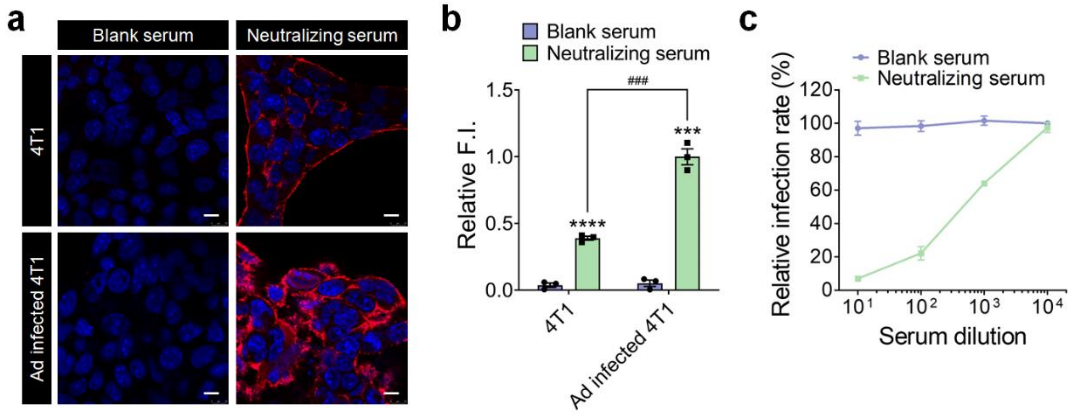

Figure S15. Ad@NKs induce immune responses against tumor and virus in 4T1-bearing mice. At day 9 post Ad@NK treatment, the serum was harvested from the 4T1-bearing mice for an immunostaining of uninfected and Ad-infected 4T1 cells. The serum from untreated tumor-free mice was used as a negative control. a) Cellular fluorescence (red) was imaged under a laser confocal microscope. Cell nuclei were stained with DAPI (blue). Scale bar: 10 μm. b) Relative F.I. of the cells. c) Relative infection rate of 4T1 cells treated with Ads which were preincubated with different serums. The infection rate by the Ads preincubated with blank serum diluted by 10^4 was set to 100%. Data are represented as mean ± SD, n = 3. $^{***}P < 0.001$; $^{****}P < 0.0001$ (vs. Blank serum); $^{###}P < 0.001$ (vs. indicated) denote significant difference.

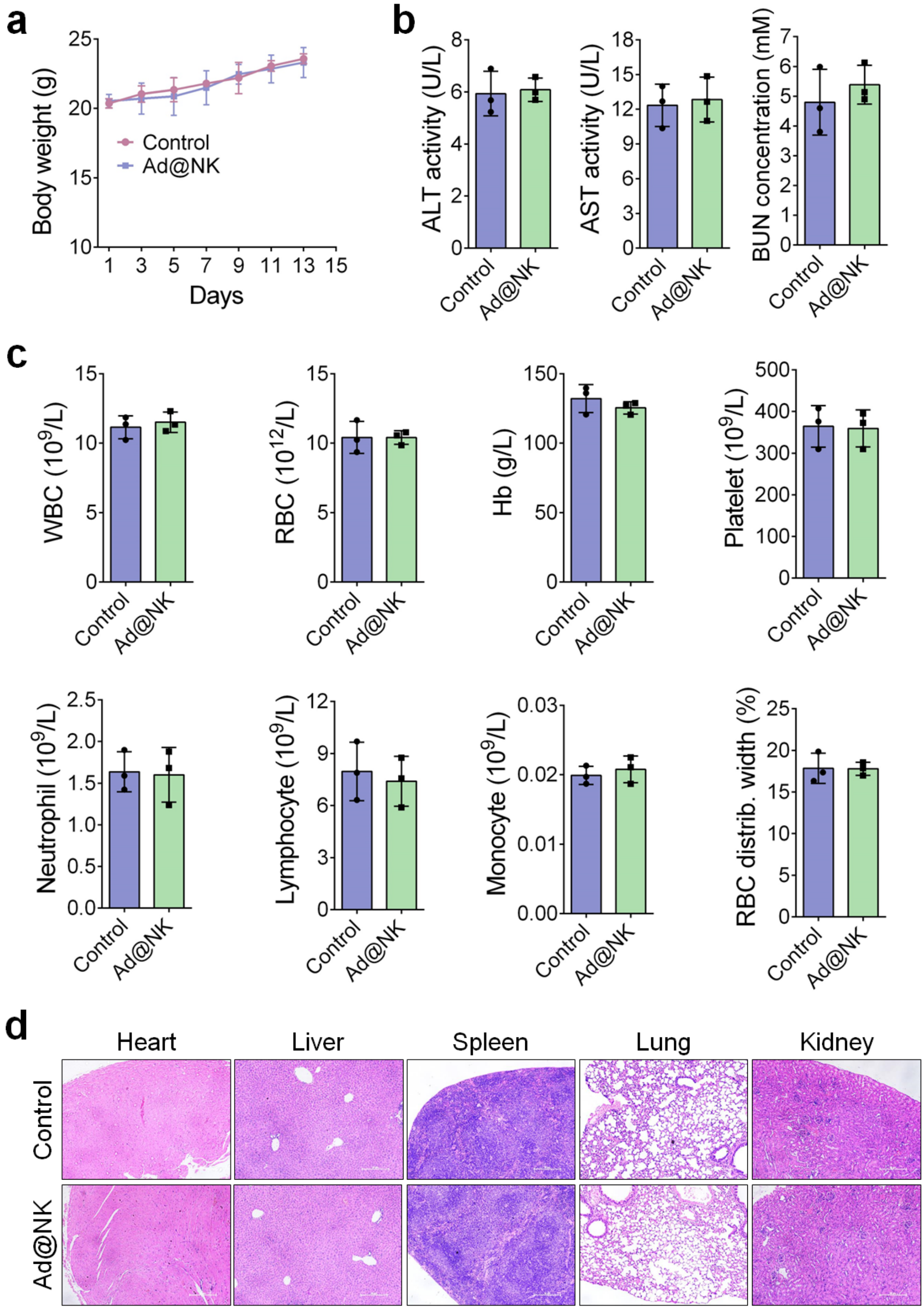

Figure S16. Safety assessment of Ad@NKs. a) Body weights of the tumor-free mice during a 14-d course of Ad@NK treatment (n = 3). b) Renal and hepatic functions and c) hematological parameters of the mice (n = 3). d) Representative photographs for the main organ tissues stained with H&E. Scale bar: 50 μm. Data are represented as mean ± SD.

Table S1 Key reagents used in this study

Reagents	Source	Identifier
Antibodies		
Mouse monoclonal anti-Adenovirus hexon protein (B315M)	Abcam	Cat# ab252760
Rabbit monoclonal anti-Calreticulin (SU37-03)	HuaBio	Cat# ET1608-60
FITC-conjugated monoclonal anti-CD11c (N418)	BioLegend	Cat# 117306; RRID: AB_313775
PE-conjugated monoclonal anti-CD40 (1C10)	Thermo Fisher	Cat# 12-0401-82; RRID: AB_465649
PE-conjugated monoclonal anti-CD80 (16-10A1)	Thermo Fisher	Cat# 16-10A1; RRID: AB_465752
PE-conjugated monoclonal anti-CD86 (GL1)	Thermo Fisher	Cat# 12-0862-82; RRID: AB_465768
FITC-conjugated monoclonal anti-CD3 (17A2)	Thermo Fisher	Cat# 11-0032-82; RRID: AB_2572431
PE-conjugated monoclonal anti-CD4 (GK1.5)	Thermo Fisher	Cat# 12-0041-82; RRID: AB_465506
APC-conjugated monoclonal anti-CD8 (53-6.7)	Thermo Fisher	Cat# 17-0081-82; RRID: AB_469335
APC-conjugated monoclonal anti-CD11b (M1/70)	BioLegend	Cat# 101211; RRID: AB_312794
PE-conjugated monoclonal anti-CD206 (C68C2)	BioLegend	Cat# 141705; RRID: AB_10896421
Rabbit polyclonal anti-IFIT1	Proteintech	Cat# 23247-1-AP; RRID: AB_2811269
Rabbit polyclonal anti-STAT4	Affinity	Cat# AF6441; RRID: AB_2835265
Rabbit polyclonal anti-phospho-STAT4 (Tyr693)	Affinity	Cat# AF3441; RRID: AB_2834883
Mouse monoclonal anti-β-actin	Beyotime	Cat# AF0003; RRID: AB_2736993
Anti-mouse IgG (H+L), HRP-linked antibody	CoWin	Cat# CW0102; RRID: AB_2736997
Anti-mouse IgG (H+L), Cy3-linked antibody	Beyotime	Cat# A0521
Anti-Rabbit IgG (H+L), HRP-linked antibody	Abbkine	Cat# A21020
Anti-Rabbit IgG (H+L), Alexa Fluor 647-linked antibody	HuaBio	Cat# HA1106
For Cell Culture		
DMEM medium	Thermo Fisher	Cat# 12430047
Alpha-MEM medium	KeyGEN	Cat# KGM11900-1
Fetal bovine serum	Thermo Fisher	Cat# 10100147
L-glutamine	Thermo Fisher	Cat# 25030149

Sodium bicarbonate	Thermo Fisher	Cat# 25080094
Inositol	Sigma Aldrich	Cat# I7508
2-mercaptoethanol	Sigma Aldrich	Cat# M3148
Folic acid	Sigma Aldrich	Cat# F8758
IL-2	Novoprotein	Cat# C013
Horse serum	Biochannel	Cat# BC-SE-HO014
Trypsin-EDTA	Thermo Fisher	Cat# 25200072
Critical Commercial Assays		
TUNEL Apoptosis Assay Kit	Beyotime	Cat# C1089
Cell Counting Kit-8	Beyotime	Cat# C0038
ECL Chemiluminescent Detection Kit	Vazyme	Cat# E411
BCA Protein Quantification Kit	Vazyme	Cat# E112
1st Strand cDNA Synthesis Kit	Vazyme	Cat# R312
SYBR qPCR Master Mix	Vazyme	Cat# Q321
Enhanced ATP Assay Kit	Beyotime	Cat# S0027
Mammalian genomic DNA extraction kit	Beyotime	Cat# D0061
Other Reagents		
DAPI	Beyotime	Cat# C1005
4% paraformaldehyde	Solarbio	Cat# P1110
Protease and phosphatase inhibitor cocktail	Beyotime	Cat# P1050
RIPA Lysis Buffer	Beyotime	Cat# P0013B
TRIzol reagent	Thermo Fisher	Cat# 15596026
Nitrocellulose membrane	GE	Cat# 10600001
Collagenase	Sigma Aldrich	Cat# C5138
Hyaluronidase	Sigma Aldrich	Cat# H3506
DNase I	Sigma Aldrich	Cat# D5025
DiR iodide	AAT Bioquest	Cat# 22070
Cy5-NHS	Meilunbio	Cat# MB12193
Hexadimethrine bromide	Beyotime	Cat# C0351

Table S2 Primers used for qRT-PCR

Primer name	Target mRNA (GenBank accession no.)	Sequence
GAPDH F	Mouse GAPDH (NM_001289726.1)	GGAGCGAGACCCCACTAACA
GAPDH R	Mouse GAPDH (NM_001289726.1)	ACATACTCAGCACCGGCCTC
IFIT1 F	Mouse IFIT1 (NM_008331.3)	CTCAGAGCAGGTCCAGTTCCTT
IFIT1 R	Mouse IFIT1 (NM_008331.3)	GGCCAGGAGGTTGTGCAT
IFIT2 F	Mouse IFIT2 (NM_008332.3)	AGTCCTCTTGGCACTGAAGCTT
IFIT2 R	Mouse IFIT2 (NM_008332.3)	TCAACCAGCGCCATTGCT
IFIT3 F	Mouse IFIT3 (NM_010501.2)	TCACATGGGCCGTCTCTCA
IFIT3 R	Mouse IFIT3 (NM_010501.2)	TTTTGGCAAACTTGTCTCACCTT
STAT4 F	Mouse STAT4 (NM_011487.5)	CATTTGCAACCCAAGGAGATG
STAT4 R	Mouse STAT4 (NM_011487.5)	TGGCAGCCCTCGTTTCC
Granzyme B F	Mouse GZMB (NM_013542.3)	CCCAGGCGCAATGTCAAT
Granzyme B R	Mouse GZMB (NM_013542.3)	CCCCAACCAGCCACATAGC
IFN-α1 F	Mouse IFNA1 (NM_010502.2)	ACCTCCACCAGCAGCTCAAT
IFN-α1 R	Mouse IFNA1 (NM_010502.2)	CCCCACCTGCTGCATCAG
EGFP F		GTCCGCCCTGAGCAAAGA
EGFP R		TCCAGCAGGACCATGTGATC
